# *Vibrio cholerae* O1 transmission in Bangladesh: insights from a nationally representative serosurvey

**DOI:** 10.1016/S2666-5247(20)30141-5

**Published:** 2020-12

**Authors:** Andrew S Azman, Stephen A Lauer, Taufiqur Rahman Bhuiyan, Francisco J Luquero, Daniel T Leung, Sonia T Hegde, Jason B Harris, Kishor Kumar Paul, Fatema Khaton, Jannatul Ferdous, Justin Lessler, Henrik Salje, Firdausi Qadri, Emily S Gurley

**Affiliations:** a Department of Epidemiology, Johns Hopkins Bloomberg School of Public Health, Baltimore, MD, USA; b Department of International Health, Johns Hopkins Bloomberg School of Public Health, Baltimore, MD, USA; c icddr,b, Dhaka, Bangladesh; d Epicentre, Paris, France; e Division of Infectious Diseases and Division of Microbiology and Immunology, University of Utah School of Medicine, Salt Lake City, UT, USA; f Division of Infectious Diseases and Division of Global Health, Massachusetts General Hospital, Boston, MA, USA; g Department of Pediatrics, Harvard School of Medicine, Boston, MA, USA; h Mathematical Modelling of Infectious Diseases Unit, Institut Pasteur, Paris, France

## Abstract

**Background:**

Pandemic *Vibrio cholerae* from cholera-endemic countries around the Bay of Bengal regularly seed epidemics globally. Without reducing cholera in these countries, including Bangladesh, global cholera control might never be achieved. Little is known about the geographical distribution and magnitude of *V cholerae* O1 transmission nationally. We aimed to describe infection risk across Bangladesh, making use of advances in cholera seroepidemiology, therefore overcoming many of the limitations of current clinic-based surveillance.

**Methods:**

We tested serum samples from a nationally representative serosurvey in Bangladesh with eight *V cholerae*-specific assays. Using these data with a machine-learning model previously validated within a cohort of confirmed cholera cases and their household contacts, we estimated the proportion of the population with evidence of infection by *V cholerae* O1 in the previous year (annual seroincidence) and used Bayesian geostatistical models to create high-resolution national maps of infection risk.

**Findings:**

Between Oct 16, 2015, and Jan 24, 2016, we obtained and tested serum samples from 2930 participants (707 households) in 70 communities across Bangladesh. We estimated national annual seroincidence of *V cholerae* O1 infection of 17·3% (95% CI 10·5–24·1). Our high-resolution maps showed large heterogeneity of infection risk, with community-level annual infection risk within the sampled population ranging from 4·3% to 62·9%. Across Bangladesh, we estimated that 28·1 (95% CI 17·1–39·2) million infections occurred in the year before the survey. Despite having an annual seroincidence of *V cholerae* O1 infection lower than much of Bangladesh, Dhaka (the capital of Bangladesh and largest city in the country) had 2·0 (95% CI 0·6–3·9) million infections during the same year, primarily because of its large population.

**Interpretation:**

Serosurveillance provides an avenue for identifying areas with high *V cholerae* O1 transmission and investigating key risk factors for infection across geographical scales. Serosurveillance could serve as an important method for countries to plan and monitor progress towards 2030 cholera elimination goals.

**Funding:**

The Bill & Melinda Gates Foundation, National Institutes of Health, and US Centers for Disease Control and Prevention.

## Introduction

As the seventh cholera pandemic approaches its 60th year, the global public health community is making plans to end cholera as a public health threat by 2030, through reducing transmission and nearly eliminating cholera mortality.^1^ However, these aspirations are challenged by new lineages of pandemic *Vibrio cholerae* O1 that repeatedly arise in south Asia and spread worldwide, seeding local and regional epidemics that can last decades, including massive contemporary outbreaks in Haiti and Yemen.^2^, ^3^, ^4^, ^5^ Without a substantial reduction in cholera transmission in highly endemic countries around the Bay of Bengal, such as Bangladesh, *V cholerae* dissemination to other parts of the world will continue, and global cholera control might never be achieved.

Despite years of research on cholera in Bangladesh, little is known about the magnitude and spatial distribution of pandemic *V cholerae* transmission across the country. Most surveillance efforts have focused on a few sites within the country, including the sentinel icddr,b hospital in Dhaka and the Demographic Health and Surveillance site in Matlab.^6^ The Ministry of Health and Family Welfare of Bangladesh is developing a national cholera control plan aimed at making substantial reductions in cholera morbidity and mortality in the next decade. With more than 164 million people living within the country, targeted interventions focusing on high-risk populations are needed. However, most patients with acute watery diarrhoea are not systematically tested for cholera, and there is no systematic reporting of suspected cholera nationally, leaving decision makers with few datapoints to devise plans or to monitor progress.

Recent advances in cholera seroepidemiology have shown that cross-sectional antibody profiles can be used to identify individuals infected by *V cholerae* O1 in the last year, thus providing a measure of the annual incidence of infection.^7^ Combined with these analytical methods, serosurveys can be used to gauge the extent of recent transmission in a population. These estimates of recent transmission can be used to prioritise resources, track progress in the fight against cholera, and identify high-risk populations for targeted interventions. Data to inform programme implementation and monitor progress would make Bangladesh's efforts to control cholera more efficient and serve as a proof of concept for a process that could be applied globally. We aimed to use samples from a nationally representative serosurvey to estimate the 2015 incidence of *V cholerae* O1 infection in Bangladesh, to identify factors associated with *V cholerae* O1 infection, and to identify areas of the country where transmission risk is the highest.

**Research in context**

**Evidence before this study**

We searched PubMed with the terms “cholera*” AND “Bangladesh” AND (“sero*” OR “incidence” OR “burden”) in the title, abstract, and keywords, restricting to publications in English after 2005. Two primary estimates of national cholera incidence were retrieved, both of which were based on assumptions that the population without access to improved sanitation in Bangladesh had the same cholera incidence (1·64 cases per 1000 people) as that measured in India in 2003. Other publications estimated the incidence of clinical cholera in discrete outbreaks and limited geographical surveillance sites, ranging from 0·2 to 4·9 clinical cholera cases per 1000 people per year. No studies described the geographical distribution of cholera incidence across Bangladesh. We found no estimates of national infection incidence. We identified no serosurveys in the general population of Bangladesh. However, one study in Dhaka showed that 30% of cholera cases were re-infected in the year after clinical disease.

**Added value of this study**

Our study provides a national estimate of *Vibrio cholerae* O1 seroincidence in Bangladesh and identifies key areas where infection risk is the highest. Since no nationally representative estimates of clinical cholera disease or infection are available, this study provides important data for decision makers to use when planning to meet the ambitious goals for elimination of cholera as a public health threat by 2030.

**Implications of all the available evidence**

While revealing the magnitude and geographical distribution of *V cholerae* O1 infection incidence in Bangladesh, our study has broader relevance in the global fight against cholera. Our study provides a model, independent of clinical surveillance systems, that other endemic countries can follow to both identify priority intervention areas and to monitor progress in the fight against cholera.

## Methods

### Study population and survey design

We used data from a previously described two-stage (household and community) cluster survey originally designed to estimate dengue seroprevalence in Bangladesh.^8^ In brief, from Oct 16, 2015, to Jan 24, 2016, 70 communities across Bangladesh (from a total of 97 162 in the 2011 national census) were selected, with probability of selection proportional to each community's population. In rural areas, communities were defined as villages, whereas in urban areas they were defined as wards (ie, neighbourhoods). Study staff identified the house at which the most recent wedding had taken place and identified the closest neighbour. They then counted six households in a random direction to identify the first household for the study. To select each additional household for the study, they used the previous household as a starting point and counted six households in a random direction. In each selected household, study staff identified the household head, described the study, and invited them to participate in the study. If the household head agreed to participate, all household members older than 6 months of age were invited to take part. Within each community, study staff continued enrolling households until at least 40 blood samples were obtained from at least ten households. Within each household, study staff administered both individual and household questionnaires with questions about household-level infrastructure, wealth and assets, and individual data on demographics and travel history, as well as obtaining 5 mL of venous blood (3 mL from children aged 3 years or younger) from all consenting individuals.

The study was approved by the icddr,b ethics review board (protocol number PR-14058); this secondary analysis was reviewed and deemed exempt from review by the Johns Hopkins Bloomberg School of Public Health Institutional Review Board. All adult participants provided written informed consent to participate in the study. Parents or guardians of all child participants provided written informed consent on their behalf.

### Laboratory methods

After collection, blood samples were centrifuged in the field, with serum extracted into separate vials before being shipped to icddr,b laboratories in Dhaka in nitrogen dry shippers (–80°C). All laboratory analyses were done at icddr,b from May to October, 2018. Vibriocidal titres were estimated with standard methods using *V cholerae* O1 Ogawa (X-25049) or Inaba (T-19479) as the target organisms, with monoclonal antibodies against lipopolysaccharide used for quality control. The complete protocol for the vibriocidal analyses, including rejection criteria and interpretation, is available online.^9^

We measured serum isotype-specific (IgM and IgG) antibody responses against *V cholerae* O1 Ogawa and Inaba lipopolysaccharide and cholera toxin B subunit using standardised ELISA methods, as previously described.^10^ We read plates kinetically at 450 nm for 5 min and normalised the maximum rate of change in optical density in milliabsorbance units per min across plates by calculating the ratio of the test sample to a monoclonal antibody standard included on each plate. ELISA samples were rejected and rerun if any of the three sample wells were greater than two times or less than half the median of the three wells.

### Statistical analysis

Our primary objective was to estimate the proportion of the population with a meaningful immunological exposure to *V cholerae* O1 within the previous year, referred to as seroincidence (or seropositive when describing the results for an individual). To estimate seroincidence, we used a previously published random forest modelling approach^7^ that identifies recently infected individuals using age, sex, vibriocidal titres (Ogawa and Inaba), IgG and IgA antibodies against lipopolysaccharide (maximum of Inaba and Ogawa for each person), and IgG and IgA antibodies against cholera toxin B subunit. The outcome of this model is a binary prediction of whether an individual with a specific antibody and demographic profile was infected in the previous year. The model was originally fitted to longitudinal data from a cohort of people in Bangladesh with confirmed cholera who had been medically attended and household contacts who were not infected. This fitted model was then validated on an external dataset of volunteers from North America who had been experimentally challenged with *V cholerae* O1 Inaba.^11^ While the most important predictor in this model is vibriocidal antibodies, which have the longest half-life of all antibodies considered, addition of other antibodies and isotypes significantly improves predictions. We not only focused on a model for identifying infections that occurred in the past year but also used similar models to make estimates of seroincidence 100 days and 200 days before sampling (appendix p 8). As a simpler (although less specific) alternative, which would allow for comparisons with historical studies, we used the vibriocidal test classifying individuals with a vibriocidal titre (maximum of Inaba or Ogawa) of 320 or higher as seropositive.^7^, ^12^

As time between sampling an individual and their last infection increases, sensitivity of the random forest model and vibriocidal tests falls (ie, the antibody signals for many people return to levels close to the preinfection baseline). We modelled this time-varying sensitivity with data from a previously published cohort study^7^ using logistic regression with a random effect for each individual and a cubic polynomial for time since infection (up to 1 year post-infection; appendix pp 3–5), similar to that used by Leisenring and colleagues.^13^ We then combined the specificity of each test and the time-varying sensitivity, assuming constant risk in the previous year (seasonal risk assessed in sensitivity analyses in the appendix pp 5–6) in a Bayesian hierarchical model, implemented in Stan,^14^ to estimate seroincidence in the surveyed population. We post-stratified estimates for each sampled community to match the 2015 age and sex distribution estimated by the WorldPop database.^15^ Full details of seroincidence models are provided in the appendix (pp 1–6).

To extend the post-stratified estimates from the sampled communities to the full population of Bangladesh, we fitted a logistic regression model to community-level seroincidence estimates from the Bayesian hierarchical model with covariates including population size,^16^ demographics (the proportion of the population that is female, aged 0–9 years, and aged 10–19 years),^15^ travel time (to the nearest city),^17^ distance to a major water body, altitude, and a poverty index,^18^ and a spatial random field assuming a Matern spatial covariance function using integrated nested Laplace approximations (INLA), as implemented in the R-INLA package.^19^ To choose the covariates for the logistic regression, we did backwards stepwise selection based on Wantabe-Akaike Information Criteria (WAIC; appendix pp 9–10).^20^ We did leave-one-out cross-validation to check the out-of-sample performance of the model that minimised WAIC. In this cross-validation, data from each community were sequentially left out of the model fitting, and the resulting model was used to predict the held-out community. We compared the mean absolute error of these predictions with those from two other models, a null model with only a Matern spatial covariance function, and a naive model that simply predicted the mean of the other communities.^21^ Using the best performing model from the leave-one-out cross validation, we estimated the joint posterior density of seroincidence at a 5 km × 5 km grid cell level across Bangladesh.^19^ We divided grid cell level estimates of seroincidence across the country by the population-weighted mean to obtain a map of relative infection risk. To estimate the number of infections in the year preceding the survey, we multiplied estimates of seroincidence by the estimated 2015 population^16^ in each grid cell. We report the posterior median and 95% credible intervals from these models (appendix pp 7–8).

We investigated associations between seropositivity and individual-level, household-level, and community-level risk factors using spatial logistic regression models with random effects for households and communities. All individual-level and household-level data were obtained directly from survey questionnaires. We included spatial correlation using a Matern covariance model and estimated the joint posterior density using INLA.^19^ Odds ratios (ORs) and corresponding 95% highest posterior density intervals (referred to as 95% CI throughout) were based on 1000 draws from the posterior. We adjusted analyses for individual-level, household-level, and community-level risk factors: sex, travel history, household income, education level of head of household, electricity in house, owns land, owns home, and urban or rural community. In sensitivity analyses, we included models that had either household-level or community-level random effects with or without the Matern spatial covariance function.

We used the GADM 3.6 spatial database for all administrative boundaries, which does not include boundary changes made after September, 2015. All analyses were done in R, version 3.6.2. Data and source code to reproduce analyses are available online,^22^ with additional details on methods and model structure provided in the appendix (pp 1–7).

### Role of the funding source

The funder had no role in study design, data collection, data analysis, data interpretation, or writing of the report. The corresponding author had full access to all data in the study and had final responsibility for the decision to submit for publication.

## Results

Between Oct 16, 2015, and Jan 24, 2016, serum samples from 2930 individuals in 707 households (median four people per household) were obtained and tested. Study participants were generally reflective of the age–sex distribution of the population, although young children were under-represented (appendix p 16). 2929 individuals in the sampled population had full vibriocidal data, of whom 571 (19%) had vibriocidal titres of 320 or higher. Applying the random forest model to 2913 individuals with complete data for all assays, age and sex, 19·9% (95% CI 15·1–25·0) of the sampled communities were estimated to have been infected with *V cholerae* O1 in the year before the survey. Extending these estimates to the full population of Bangladesh, accounting for spatial correlation in risk, the national seroincidence was estimated to be 17·3% (95% CI 10·5–24·1), with alternative models, including those accounting for seasonality of infection risk, producing similar results (appendix pp 7–8).

**Figure 1:**
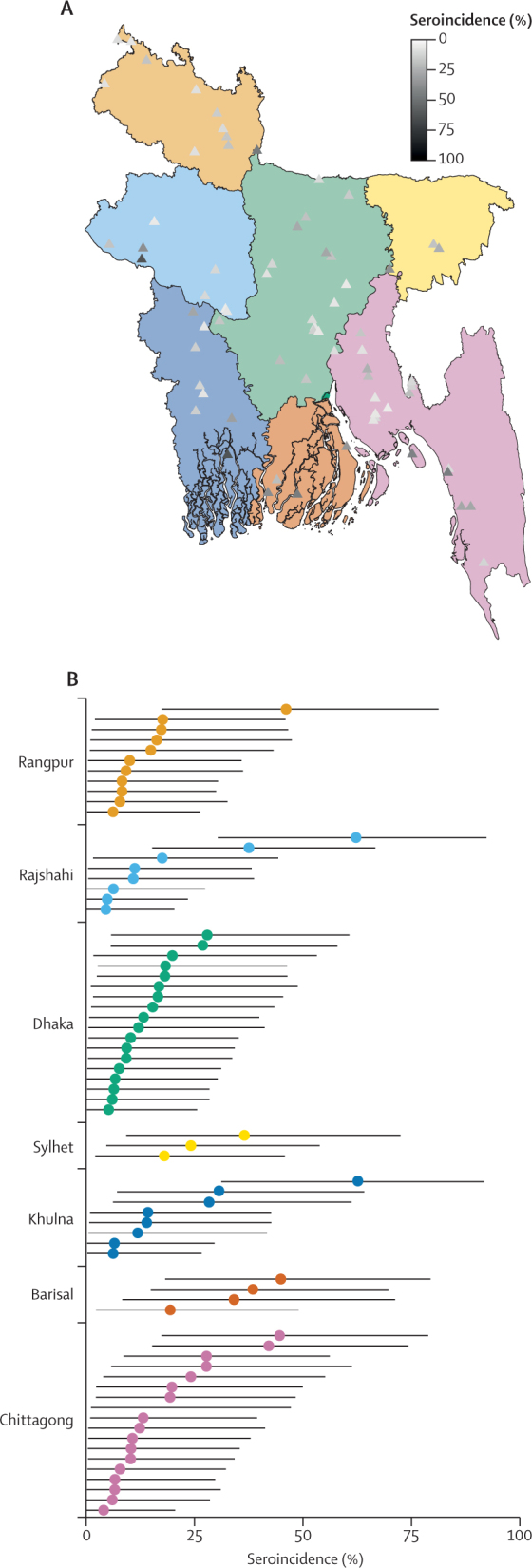
Location and seroincidence of sampled communities, by division Map shows the location of each community, with different colours representing the seven divisions of Bangladesh and triangle shading representing the median seroincidence estimate (A). Plot shows seroincidence estimates and accompanying 95% credible intervals for each community, grouped by division (B); colours accord with those in (A).

Within households, seroincidence ranged from 0% to 100%, with 339 (48%) of 707 households having no individuals who were seropositive. Members of households with at least one other individual who was seropositive had a 1·70-fold (95% CI 1·46–1·98) increase in the risk of being seropositive compared with those with no other individuals in the household who were seropositive. Seroincidence varied across sampled communities, with two of 70 communities having estimated seroincidence less than 5% (minimum 4·3%) and two communities with seroincidence of more than 50% (maximum 62·9%; figure 1). Clustering of seropositivity within households (intraclass correlation coefficient [ICC] 39·9%, 95% CI 19·2–62·5) and within communities (34·2%, 16·4–56·9) accounted for most variation in seroincidence estimates (combined ICC 76·0%, 95% CI 52·2–92·9).

The table shows associations between seropositivity and individual-level, household-level, and community-level risk factors. Children younger than 10 years had two times lower odds of seropositivity than did individuals aged 20 years and older (adjusted OR 0·49, 95% CI 0·34–0·70). No difference was noted between men and women (adjusted OR 1·11, 95% CI 0·91–1·35), and no association with seropositivity was seen with recent travel history. While population size was not associated with seropositivity, in unadjusted analyses, individuals living in areas classified as urban by the 2011 census had 36% increased odds of being seropositive (OR 1·36, 95% CI 1·00–1·83). Alternative models, including univariate models and those without spatial correlation, led to similar estimates of the relation between cholera risk and potential risk factors (appendix p 15).

**Table:** Estimated associations between individual-level, household-level, and community-level factors and seropositivity from spatial logistic regression models

		Patients (n=2901)*	**Full model, adjusted odds ratio (95% CI)**	**Univariate model, unadjusted odds ratio (95% CI)**
**Individual-level factors**				
Age, years				
	0–9	337 (12%)	0·49 (0·34–0·70)	0·49 (0·34–0·69)
	10–19	745 (26%)	0·71 (0·56–0·89)	0·73 (0·58–0·91)
	≤20	1819 (63%)	1 (ref)	1 (ref)
Sex				
	Male	1391 (48%)	1·11 (0·91–1·35)	1·07 (0·89–1·28)
	Female	1510 (52%)	1 (ref)	1 (ref)
Travel history				
	No travel in past 6 months	1215 (42%)	1 (ref)	1 (ref)
	Travel in past week	444 (15%)	1·15 (0·83–1·60)	1·17 (0·88–1·57)
	Travel in past month	593 (20%)	0·91 (0·68–1·22)	0·97 (0·74–1·26)
	Travel in past 6 months	649 (22%)	1·01 (0·77–1·32)	1·05 (0·82–1·34)
**Household-level factors**				
Household income per month, US$†				
	<90	309 (11%)	1·41 (0·94–2·11)	1·25 (0·91–1·71)
	91–130	534 (18%)	1·02 (0·74–1·42)	0·99 (0·76–1·30)
	131–261	1094 (38%)	1 (ref)	1 (ref)
	>261	964 (33%)	0·95 (0·71–1·27)	0·92 (0·72–1·16)
Education (head of household)				
	No school	900 (31%)	1 (ref)	1 (ref)
	Primary school	746 (26%)	1·21 (0·89–1·66)	1·17 (0·91–1·51)
	Secondary school	791 (27%)	1·09 (0·80–1·49)	1·08 (0·84–1·39)
	Post-secondary education	464 (16%)	0·77 (0·52–1·15)	0·77 (0·56–1·05)
Electricity in house		2629 (91%)	1·17 (0·76–1·81)	1·07 (0·76–1·51)
Owns land		2315 (80%)	0·99 (0·72–1·36)	0·94 (0·74–1·21)
Owns home		2717 (94%)	1·04 (0·59–1·83)	0·94 (0·60–1·47)
**Community-level factors**				
Urban		733 (25%)	1·51 (0·94–2·44)	1·36 (1·00–1·83)
Distance to major water body, per 10 km		1·00 (1·17)	0·90 (0·76–1·07)	0·91 (0·78–1·06)
Poverty index		−0·10 (0·59)	1·04 (0·57–1·93)	1·14 (0·85–1·53)
Travel time to nearest city, min		12·63 (14·57)	0·99 (0·98–1·01)	0·99 (0·98–1·00)
Altitude, m		16·78 (16·12)	1·00 (0·98–1·01)	0·99 (0·98–1·01)
Population, log		10·72 (1·19)	0·86 (0·66–1·13)	1·00 (0·87–1·14)

Data are n (%) or mean (SD), unless otherwise indicated. The full model includes all covariates shown in the table, random effects for household and community, in addition to a Matern spatial correlation function.

* Patients with complete data for all variables.

† Categories in Bangladesh Taka (TK) are <7000, 7000–9999, 10 000–20 000, and >20 000; TK77·6=US$1 (June, 2015).

**Figure 2:**
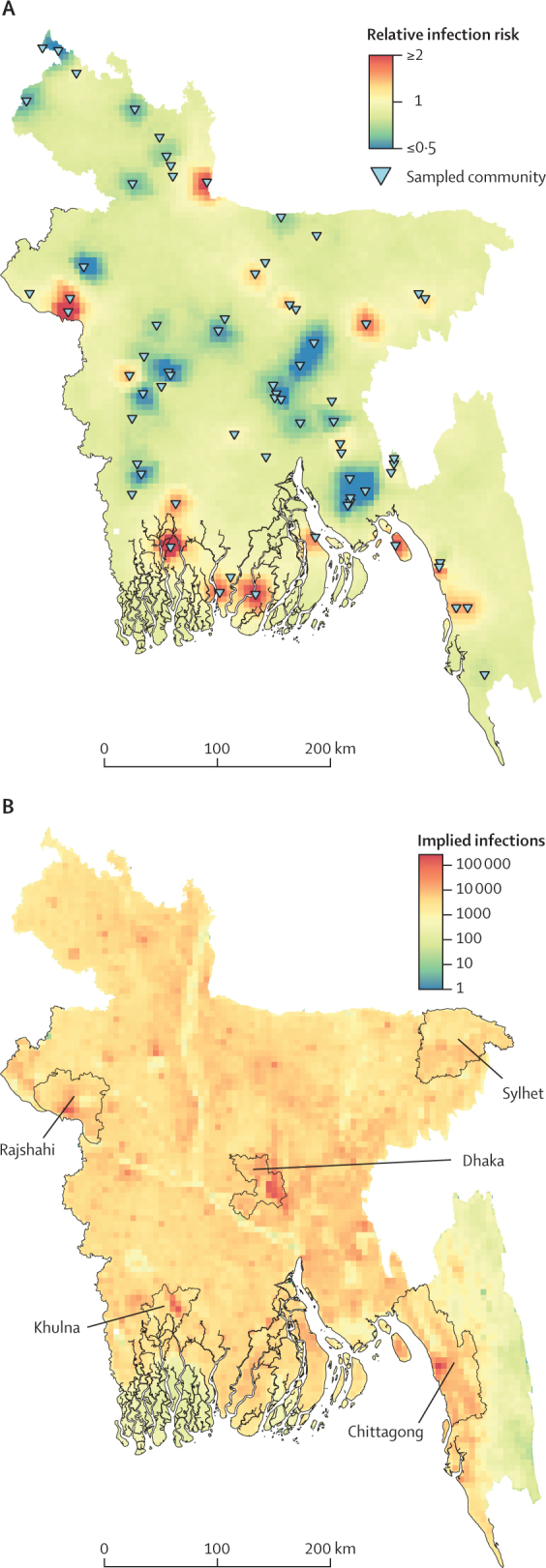
Relative risk of *Vibrio cholerae* O1 infection and estimates for the number of annual infections Maps show the relative risk estimated at a 5 km × 5 km grid cell level (A) and infection estimates per 5 km × 5 km grid cell (B) across Bangladesh. Sampled sites are shown in triangles (A). The districts containing the five most populous cities in Bangladesh are labelled (B). The relative risk for each grid cell is estimated by applying integrated nested Laplace approximations with a Matern spatial covariance model to post-stratified predictions of sampled communities from a random forest model that are corrected for demographics (age and sex), the sampling design of the study, and sensitivity and specificity using a Bayesian hierarchical model.

Our maps show heterogeneity of *V cholerae* O1 infection risk, with community-level (ie, grid cell) relative infection risks ranging from 0·34 to 3·02 (figure 2A). The highest risk of infection in Bangladesh was seen around the Bay of Bengal, including the peninsula, with pockets of high relative risk estimated in the central-west region (eg, parts of Rajshahi), central-east region (eg, parts of Sylhet), and north of the country (eg, parts of Rangpur). When aggregated to the district-level, only three districts (Borgona, Khulna, and Rajshahi; appendix pp 12–14) had a seroincidence 20% or higher than the national average, with substantial uncertainty. Chittagong and Nasirabad (now known as Mymensingh) districts each had more than 1 million estimated infections. Relative to other areas of Bangladesh, the annual risk of infection in Dhaka district was 82·4% (95% CI 31·6–151·7) of the average national risk (median seroincidence 14·4%, 95% CI 4·5–28·4). 28·1 (95% CI 17·1–39·2) million *V cholerae* O1 infections were estimated to have occurred in the year before the survey, with annual infections of more than 2·0 (0·6–3·9) million in Dhaka, constituting 7·0% (95% CI 2·7–12·8%) of all infections in Bangladesh (figure 2B; appendix pp 12–14).

## Discussion

Our findings show that, across Bangladesh, about one in six people were infected with *V cholerae* O1 in the year before the serosurvey, with variability in risk between households and communities. Our serology-based approach to estimating the magnitude of transmission and geographical variability in cholera risk illustrates a new avenue for understanding cholera epidemiology without the traditional biases related to care-seeking behaviour and imperfect reporting.

Our results highlight the differences between relative risk and absolute infection burden in Bangladesh. Dhaka is typically regarded as one of the highest risk areas for cholera in Bangladesh, perhaps because the icddr,b reference cholera hospital is located in the city, and many cholera-related research studies have been done there.^23^ Prioritising cholera control interventions in Dhaka might be justified, despite the city not having the highest relative risk, because of Dhaka's role as a major transport and migration hub and the large number of infectious people within the city. Despite having small populations, Chittagong and Nasirabad districts each had more than 1 million infections. Borgona, Khulna, and Rajshahi had the highest estimated seroincidence (and relative risk) of any district, although our power to precisely estimate seroincidence in small geographical areas was limited by our sample size.

Serosurveys have several limitations related to generalisability and public health use. The year before the study was done (roughly the 2015 calendar year) seemed to be an average year for floods and precipitation, according to the Bangladesh Flood Forecasting and Warning Centre, which are both factors that could modify cholera risk. Based on limited data from clinical sentinel surveillance, cholera case reports in 2015 were consistent with historic trends.^24^ However, our estimates might not generalise to all future years because of natural variation in infection incidence driven by both intrinsic and extrinsic factors. Among the individual-level, household-level, and community-level covariates considered by us, only age was a strong predictor of risk for seropositivity. Our data were not originally obtained for study of cholera, therefore, we did not have many potentially informative covariates (eg, those related to water and sanitation) and other factors (eg, historical reports of severe diarrhoea).

As with any diagnostic test, precision of our seroincidence estimates is limited by the sensitivity and specificity of the random forest model. Our model was trained on data from a cohort of individuals with severe cholera and uninfected contacts in Bangladesh, with few young children and no individuals older than 60 years. Vibriocidal antibody kinetics seem to decay faster in children than in adults,^7^, ^25^ which could lead to underestimation of seroincidence among young children in our study. However, in view of the relatively similar proportion of children across most of Bangladesh,^15^ we do not expect this potential limitation of differential antibody kinetics to have major effects on any relative results (eg, comparing seroincidence between areas). Future studies describing the post-infection kinetics of mild and asymptomatic *V cholerae* infections, in addition to infections in young children and older people, will help improve the generalisability of this recent infection model.

Our estimates of seroincidence are high but accord with findings of previous longitudinal household studies in Bangladesh.^26^, ^27^, ^28^ For example, a study in Dhaka followed up individuals with confirmed cholera serologically after infection and found that 30% had evidence of re-infection in the subsequent year.^26^ Published estimates of the proportion of infections that result in clinical disease range widely, with some estimates suggesting more than 100 *V cholerae* infections for each severe cholera case and others suggesting four infections per severe case.^12^, ^29^, ^30^ This ratio probably varies by infecting strain, setting, and the ingested inoculum. Future studies gathering clinical surveillance, health-seeking behaviour, and serology data across areas with different epidemiological and transmission characteristics can help us better estimate the true distribution of this ratio and help translate estimates of infection incidence to estimates of clinical disease and public health burden.

These nationally representative data provide an opportunity to gain new insights to guide the design of future serosurveys related to cholera. Although the raw antibody results primarily drive our estimates of seroincidence, corrections for test performance based on time since infection can have a substantial effect. Depending on the primary objective of a serosurvey, models and sampling design can be tailored to maximise test performance. For example, if the aim is to estimate the true size of an outbreak, doing a serosurvey just after an outbreak, or serial serosurveys during protracted outbreaks, can lead to increased sensitivity and more precise estimates of risk. Because of its moderate specificity, this approach is unlikely to verify the absence of cholera transmission in an area. Even if tuned to maximise specificity, the lower limits of detection would be well above one infection per 1000 population, which is too high in most settings to declare a community cholera-free. Addition of new markers that can help discriminate between proximal and distal infections might improve model performance and bring specificity closer to 100% while maintaining adequate sensitivity.

In summary, our nationally representative serosurvey highlights the high proportion of Bangladesh's population infected annually with *V cholerae* O1 and identifies potential target areas, both populations with higher risk for infection and higher numbers of total infections, for efficient deployment of control measures, including vaccines. Serosurveillance can be an important approach for tracking changes in transmission, and the effect of interventions, over the coming years in Bangladesh and globally. Efforts to improve the ease of deploying these studies and testing of samples would further increase their value.
