# Supplementary material for: Vibrio cholerae O1 transmission in Bangladesh: insights from a nationally representative serosurvey
Source: Lancet Microbe. 2020 Dec;1(8):e336–43. doi: 10.1016/S2666-5247(20)30141-5 (PMC7738617; doi:10.1016/S2666-5247(20)30141-5)
Supplement: Supplementary appendix [file mmc1.pdf]

THE LANCET
Microbe

Supplementary appendix

This appendix formed part of the original submission and has been peer reviewed. We post it as supplied by the authors.

Supplement to: Azman A S, Lauer S A, Bhuiyan T R, et al. *Vibrio cholerae* O1 transmission in Bangladesh: insights from a nationally representative serosurvey. *Lancet Microbe* 2020; **1:** e336–43.

Vibrio Cholerae O1 Transmission in Bangladesh: Insights from a Nationally-Representative Serosurvey Supplement

Andrew S Azman[a,*], Stephen A Lauer[a,*], M. Taufiq Rahman Bhuiyan[b], Francisco J Luquero[c,d], Daniel T Leung[e], Sonia Hegde[a], Jason Harris[f,g,h], Kishor Kumar Paul[b], Fatema Khaton[b], Jannatul Ferdous[b], Justin Lessler[a], Henrik Salje[i,a,**], Firdausi Qadri[b,**], Emily S Gurley[a,b,**]

[a] *Department of Epidemiology, Johns Hopkins Bloomberg School of Public Health, Baltimore, USA*
[b] *icddr,b, Dhaka, Bangladesh*
[c] *Epicentre, Paris, France*
[d] *Department of International Health, Johns Hopkins Bloomberg School of Public Health, Baltimore, USA*
[e] *Division of Infectious Diseases, University of Utah School of Medicine, Salt Lake City, USA*
[f] *Division of Infectious Diseases, Massachusetts General Hospital, Boston, USA*
[g] *Division of Global Health, Massachusetts General Hospital, Boston, USA*
[h] *Department of Pediatrics, Harvard School of Medicine, Boston, USA*
[i] *Mathematical Modelling of Infectious Diseases Unit, Institut Pasteur, Paris, France*

S1. Cholera seroincidence model and inference

The primary goal of these analyses is to estimate the proportion of the population infected by *Vibrio cholerae* O1 in the previous year, which we refer to as the 'seroincidence rate' and denote as π. We use a previously validated random forest model to classify whether each member of a recent nationally-representative serosurvey was infected in the year before the before the survey. We treat the binary outcome of this model like a diagnostic test, which when summarized at the population-level can be adjusted for sensitivity and specificity. As detailed in the paper, the serosurvey was a two-stage cluster survey, with 70 communities selected with probability proportional to each community's population and at least 10 households (with at least 40 total samples) sampled from each community.

We make three estimates of the seroincidence rate, all of which rely on a Bayesian hierarchical model that is specified below. For the 'survey estimate', we assume that the nationwide estimate of seroincidence is equivalent to the in-sample estimate of seroincidence, since the serosurvey sample was nationally representative. For the 'post-stratified estimate', we use the parameter estimates from the model to predict the seroincidence in the sampled communities based on their demographics. For the 'spatial estimate', we extend the post-stratified estimate to the rest of the country using a logistic regression model including covariates and a Matern spatial covariance function.

S1.1. Bayesian hierarchical model

We model the random forest predictions of seropositivity (see Section S1.3) for each individual, z_i ($i = 1, \dots, n$, from household $h = 1, \dots, H$ in community $c = 1, \dots, C$), with a Bayesian hierarchical model similar to that of Makela, Si, and Gelman,[1] augmented to account for the sensitivity and specificity of the random forest model:

$$z_i \sim \text{Bernoulli}(\pi_i \theta^{1|1} + (1 - \pi_i)(1 - \theta^{0|0})) \tag{1}$$

$$\text{logit}(\pi_i) = \alpha_{c_i} + \alpha_{h_i} + \mathbf{X}_i \boldsymbol{\beta} \tag{2}$$

$$\alpha_c \sim \text{Normal}(\alpha_0 + \gamma \log(N_c), \sigma_c^2) \tag{3}$$

$$\alpha_h \sim \text{Normal}(0, \sigma_h^2) \tag{4}$$

*Co-first authors
**Co-last authors

We separate the probability that an individual is predicted to be seropositive by the random forest model into two parts: the true positive rate, where the underlying probability of being seropositive π_i is multiplied by the sensitivity $\theta^{1|1}$, and the false positive rate, calculated as the underlying probability of being seronegative $(1 - \pi_i)$ multiplied by one minus the specificity $(1 - \theta^{0|0})$.

We assume that the underlying probability of being seropositive π_i for each individual is logit-normal with random effects for their community α_{c_i} and household α_{h_i} and fixed effects $\boldsymbol{\beta}$ for their age and sex $\mathbf{X}_i$. The community-level random effect has a mean determined by the sum of a country-level intercept, α_0, and linear term to account for the (log) population of the community (N_{c_i} with coefficient γ) and a variance σ_c^2. Since the probability that a community was sampled was proportional to its population, we need to account for any relationship between community population and seroincidence rate to control for confounding. The household-level random effect is centered at zero with a variance of σ_h^2. We use weak standard normal priors for α_0, γ, and the β coefficients and t distributions truncated to be positive as priors for σ_c and σ_h. Section S1.4 has more details about estimating the sensitivity and specificity.

Upon estimating π_i, we can estimate the seroincidence of each community π_c^{survey} and the whole survey π^{survey}:

$$\hat{y}_i = \text{Bernoulli}(\pi_i) \tag{5}$$

$$\pi^{survey} = \frac{1}{n}\sum_{i=1}^{n}\hat{y}_i \tag{6}$$

$$\pi_c^{survey} = \frac{\sum_{i\in c}\hat{y}_i}{\sum_{i\in c} n_i}. \tag{7}$$

The serostatus of each individual is determined by a draw from Bernoulli with probability π_i. These draws can be averaged across each community to calculate the community-specific survey estimates π_c^{survey} or across all individuals to calculate the survey estimate π^{survey}.

For each sampled community, we estimated the seroincidence $\pi_c^{poststrat}$ by post-stratifying the posterior samples of our parameter estimates to match the demographics of that community. For every combination of age category ($a = 1, \ldots, A = 3$), sex ($s = 0, 1$), and community c, we estimated the probability of seropositivity, $\pi_{a,s,c}$ for each posterior draw of $\boldsymbol{\beta}$, α_c, and σ_h^2. We can estimate the community seroincidence $\pi_c^{poststrat}$ by taking a weighted average of the $\pi_{a,s,c}$, where weights are determined by the demographic distribution of the community.

$$\pi_{a,s,c} = \int_0^1 logit^{-1}(\alpha_c + \mathbf{X}_{a,s,c}\boldsymbol{\beta} + \sigma_h * \Phi^{-1}(t))dt \tag{8}$$

$$\pi_c^{poststrat} = \sum_{a=1}^{A}\sum_{s=0}^{1}\frac{N_{a,s,c}\pi_{a,s,c}}{N_c} \tag{9}$$

$$\pi^{poststrat} = \frac{1}{C}\sum_{c=1}^{C}\pi_c^{poststrat} \tag{10}$$

where $\Phi^{-1}(t)$ is the quantile function of a standard normal distribution. Since communities were chosen at random (weighted by population), we assume that a simple average of the sampled communities is sufficient for estimating the overall post-stratified seroincidence $\pi^{poststrat}$.

We fit the Bayesian hierarchical model in the Stan probabilistic programming language and used the `rstan` package in R to run the model and analyse outputs. We ran 5,000 iterations (4 chains with 1,500 iterations each with 250 for warm-up) and assessed convergence visually and using the R-hat statistic.[2,3]

S1.2. Integrated nested Laplace approximations and the spatial estimate

Our primary estimate of the country-wide seroincidence used in the main analyses is the spatial estimate. To produce this estimate, we extend the community-specific post-stratified estimates to the entire country using a logistic regression model with a Matern spatial covariance function and covariates with integrated nested Laplace approximations (INLA),[4] as described in the main text. To choose the covariates, we conduct a backwards stepwise regression, based on Wantabe-Akaike Information Criteria (WAIC), where the outcome

is the average seroincidence in each sampled community. The covariates considered were log population, proportion female, proportion age 0-9, proportion age 10-19, distance to major body of water, a poverty index, travel time to a major city, and altitude. After step-wise selection, we compared the predictive skill of the best model with covariates to a null model with only a Matern spatial covariance function (no covariates) by conducting a leave-one-community-out cross validation and evaluating the results with mean absolute error (MAE). To make the nationwide estimates, we fit the INLA model with the lowest MAE to each of 1,000 posterior draws of community seroincidence from the Bayesian hierarchical model (described above) and then predict the seroincidence for all 5km by 5km grid-cells across the country. In the end, we generate 1,000 maps of cholera seroincidence rates and we take a population-weighted average to produce the nationwide spatial estimates. Our primary results are the median spatial estimate and the 95% credible interval.

S1.3. Random forest predictions

Azman *et al.* fitted a random forest model using age, sex, vibriocidal titers (Ogawa and Inaba), anti-LPS IgG and IgA antibodies, and anti-CTB IgG and IgA antibodies, and blood group to classify the seropositive status of individuals from a longitudinal cohort study in Bangladesh.[5] Since no blood group information was collected in the serosurvey, we fit a new random forest model to the cohort data using all of the remaining covariates. For each observation in the cohort study, we use the proportion of trees that predict that the observation is seropositive as the probability of seropositivity. From these probabilities, we calculate the receiver operating characteristic (ROC) curve for the cohort predictions and calculate the cutoff that maximizes the Youden's J statistic, i.e. the sum of the sensitivity and the specificity.[6]

We use the random forest model to predict the seropositivity status of each participant in the serosurvey; the participants whose probability of seropositivity exceed the Youden cutoff are classified as seropositive, z_i in Equation 1.

S1.4. Specificity and sensitivity of the random forest predictions

As with all imperfect tests, population-level (e.g., aggregated) random forest model seroincidence estimates can be corrected for the test's specificity and sensitivity, when known. To estimate the specificity and sensitivity of this random forest model we conducted leave-one-individual-out cross validation (LOOCV) on the original cohort data used in Azman *et al.*. Each participant j ($j = 1, \ldots, J$) was sampled at multiple times t, where the recent infection status $y_{j,t}^{cohort}$ was known For each individual in the cohort, we fit a random forest model to the rest of the cohort, calculate the Youden cutoff, and predict the seropositivity for all of the timepoints of the left-out individual, which we call LOOCV predictions and denote $z_{j,t}^{cohort}$.

To estimate the specificity, $\theta^{0|0}$ in Equation 1, we include the LOOCV predictions in our Bayesian hierarchical model:

$$[z_{j,t}^{cohort} \mid y_{j,t}^{cohort} = 0] \sim \text{Bernoulli}(1 - \theta_j^{0|0}) \tag{11}$$

$$\text{logit}(\theta_j^{0|0}) \sim \text{Normal}(\alpha_j^{\theta^{0|0}}, \sigma^2_{\theta^{0|0}}) \tag{12}$$

$$\theta^{0|0} = \frac{1}{J}\sum_{j=1}^{J} \theta_j^{0|0} \tag{13}$$

We found that there was correlation between the LOOCV predictions of the seronegative observations within an individual, but that there was no effect of time since infection.[1] We model these predictions as Bernoulli random variables with the probability of seropositivity equal to $(1 - \theta_j^{0|0})$, where the individual specificity $\theta_j^{0|0}$ is distributed logit-normal with a mean $\alpha_j^{\theta^{0|0}}$ and standard deviation $\sigma^2_{\theta^{0|0}}$.

The sensitivity of the random forest predictions varies across days since infection due to the decay in antibody response over time, while specificity remains constant given that it is defined by looking at test performance in uninfected individuals. After infection with *V. cholerae* O1, most antibodies rise including vibriocidals, one of the most informative markers, which peak around 7-10 days post-infection. As time

[1]Furthermore, some times since last *V. cholerae* infection were unknown, such as baseline measurements for infected individuals (where vibriocidal titers had yet to rise) and for the household contacts (who had low vibriocidal titers at all time points).

since infection increases, the antibody profile of an individual, in general, returns to pre-infection levels. The vibriocidal titers decay quickly in the first three months before decaying more slowly over the following three years. This decay is illustrated by the decline in raw sensitivity of the random forest predictions over time (Table S1).

Table S1: The estimated sensitivity of the random forest predictions of seropositivity over days since infection based on the Bangladesh cohort data.

Days since infection	Observations	Sensitivity
7-10	311	96.8%
24-41	293	97.3%
76-109	164	72.0%
154-199	137	46.7%
261-274	42	38.1%
353-363	37	32.4%

To account for this decay, we estimate the sensitivity as a time-varying quantity rather than as a static quantity and rewrite the overall sensitivity as a joint probability:

$$\underbrace{\theta^{1|1}}_{\substack{\text{overall}\\ \text{sensitivity}}} = \sum_{t=1}^{365} \underbrace{\mathbb{P}(Z=1 \mid Y=1, T=t)}_{\text{time-varying sensitivity}} \underbrace{\mathbb{P}(T=t \mid Y=1)}_{\substack{\text{daily probability}\\ \text{of infection}}}, \tag{14}$$

where Z is the result of the test (i.e. the random forest model), Y is the true seropositive status of the individual, and T is the time since infection in days. We need to estimate the time-varying sensitivity, $\mathbb{P}(Z=1 \mid Y=1, T=t)$, and the probability of being infected $T=t$ days ago, $\mathbb{P}(T=t \mid Y=1)$. Since sensitivity only concerns seropositive individuals (i.e. $Y=1$) and seropositivity is by our definition infection over the past 365 days, T is restricted to be less than or equal to 365 days for all components of $\theta^{1|1}$.

S1.4.1. Time-varying sensitivity

We estimate the time-varying sensitivity of the random forest predictions in the cohort study using a logistic regression model with a random coefficient for each individual $\alpha_j^{\theta^{1|1}}$ and a cubic polynomial for the log of days since infection, similar to the method used by Leisenring *et al.*:[7]

$$[z_{j,t}^{cohort} \mid y_{j,t}^{cohort} = 1] \sim \text{Bernoulli}(\theta_j^{1|1}(t)) \tag{15}$$

$$\text{logit}(\theta_j^{1|1}(t)) = \alpha_j^{\theta^{1|1}} + \beta_1 \log(t) + \beta_2 \log(t)^2 + \beta_3 \log(t)^3 \tag{16}$$

$$\alpha_j^{\theta^{1|1}} \sim \text{Normal}(\alpha_0^{\theta^1|1}, \sigma^2_{\theta^{1|1}}) \tag{17}$$

$$\mathbb{P}(Z=1 \mid Y=1, T=t) = \frac{1}{J}\sum_{j=1}^{J} \theta_j^{1|1}(t) \tag{18}$$

The posterior median and 95% credible interval for the sensitivity at each time since infection from 5 to 365, $\mathbb{P}(Z=1 \mid Y=1, T=5, \ldots, 365)$, is shown Figure S1. Days 1 through 4 were excluded as the serological biomarkers for cases had yet to rise and thus tests conducted during this time frame were considered negative baseline measurements as in Azman *et al.*

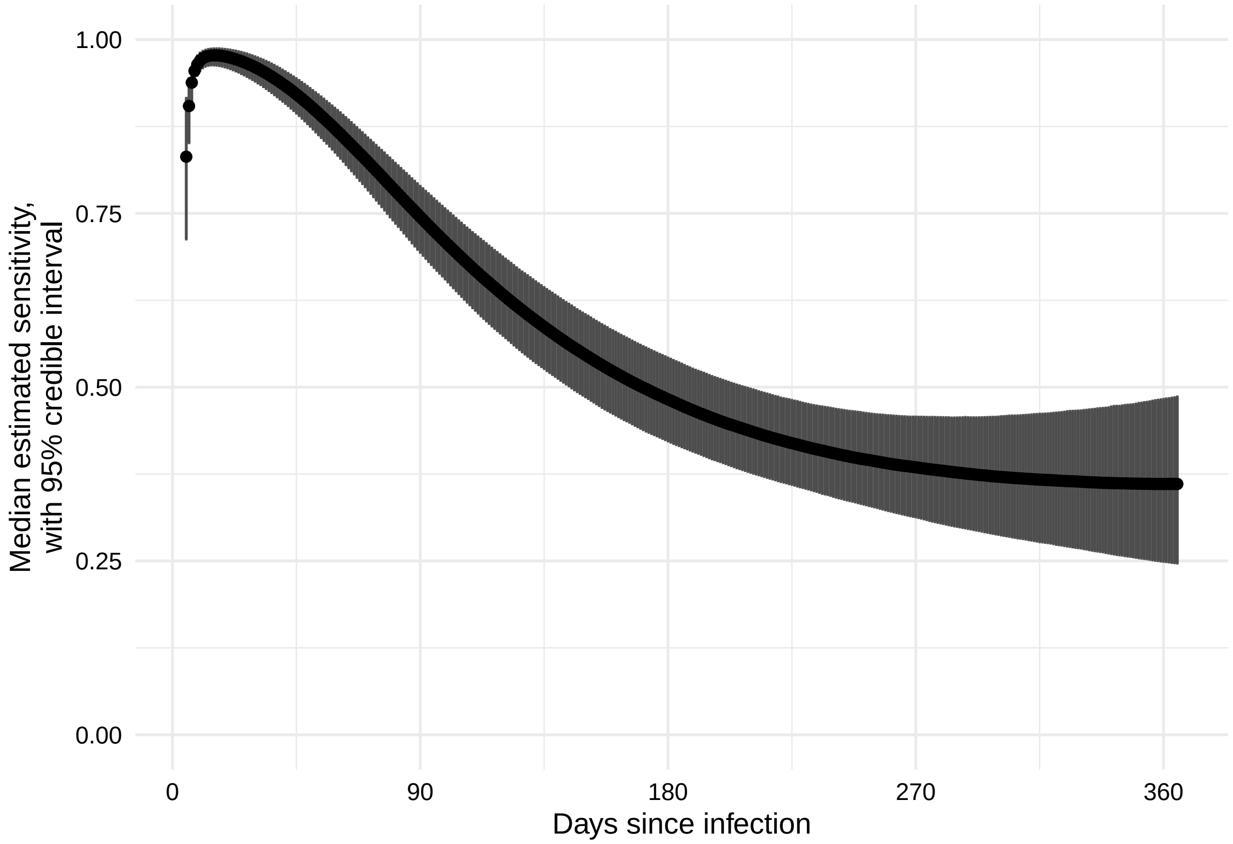

Figure S1: The estimated sensitivity of the random forest model for identifying whether an individual was infected in the last year by the number of days since V. cholerae infection. The points represent the median estimate from a generalized logistic regression model for sensitivity with cubic polynomial terms for the log of days since infection. The gray error bars represent the 95% credible intervals.

S1.4.2. Daily probability of infection

The estimates of time-varying sensitivity allow us to calculate the overall sensitivity given the time since infection, however we do not know this time for any individual in the serosurvey. We assume that individuals only get infected once in the past year, such that the daily probabilities sum to one across $T = 1, \ldots, 365$. In our primary analyses we assume that the risk of infection for each individual was uniformly distributed over the year before sample collection:

$$\mathbb{P}(T = 1 \mid Y = 1) = \mathbb{P}(T = 2 \mid Y = 1) = \cdots = \mathbb{P}(T = 365 \mid Y = 1) = \frac{1}{365}. \quad (19)$$

We set the expected value of any given time since infection to be equal to $\frac{1}{365}$, however the Bayesian hierarchical model allows for variability around each estimate.

S1.4.2.1. Alternate analysis: incorporating seasonality. Past work on clinical cholera has shown that there is seasonal variation of cholera in Bangladesh, which varies regionally across the country.[8] To estimate the probability of infection by day for each sampled community $\mathbb{P}(T = t \mid Y = 1, C = c)$, we combine sentinel surveillance testing data from Khan *et al.* (the proportion of acute watery diarrhea (AWD) cases that are cholera)[9] with district-level acute watery diarrhea data from Hegde *et al.* (in prep).

We estimate the monthly proportion of AWD cases that are cholera at each serosurvey site, using a logistic generalized additive model with a thin-plate spline for longitude and latitude, a cubic cyclic spline for month, and a random effect for year (from 2014-2016) as implemented in the brm package in R. These estimates are aggregated to the district-level (second administrative unit), where they can be multiplied by the number of AWD cases per district to calculate the number of cholera cases per district per month. For

each district that contains a sampled community, we fit a model with a monotonically increasing p-spline for the cumulative number of cholera cases from the beginning of 2014 through 2016, with the number of cumulative cases assigned to the last day of each month. From this model, we can predict the daily number of cholera cases for each location for the 365 days preceding the serosurvey for each sampled community (Figure S2). We assume that the daily probability of infection is proportional to the daily number of cases and that a person can only be infected once per year.

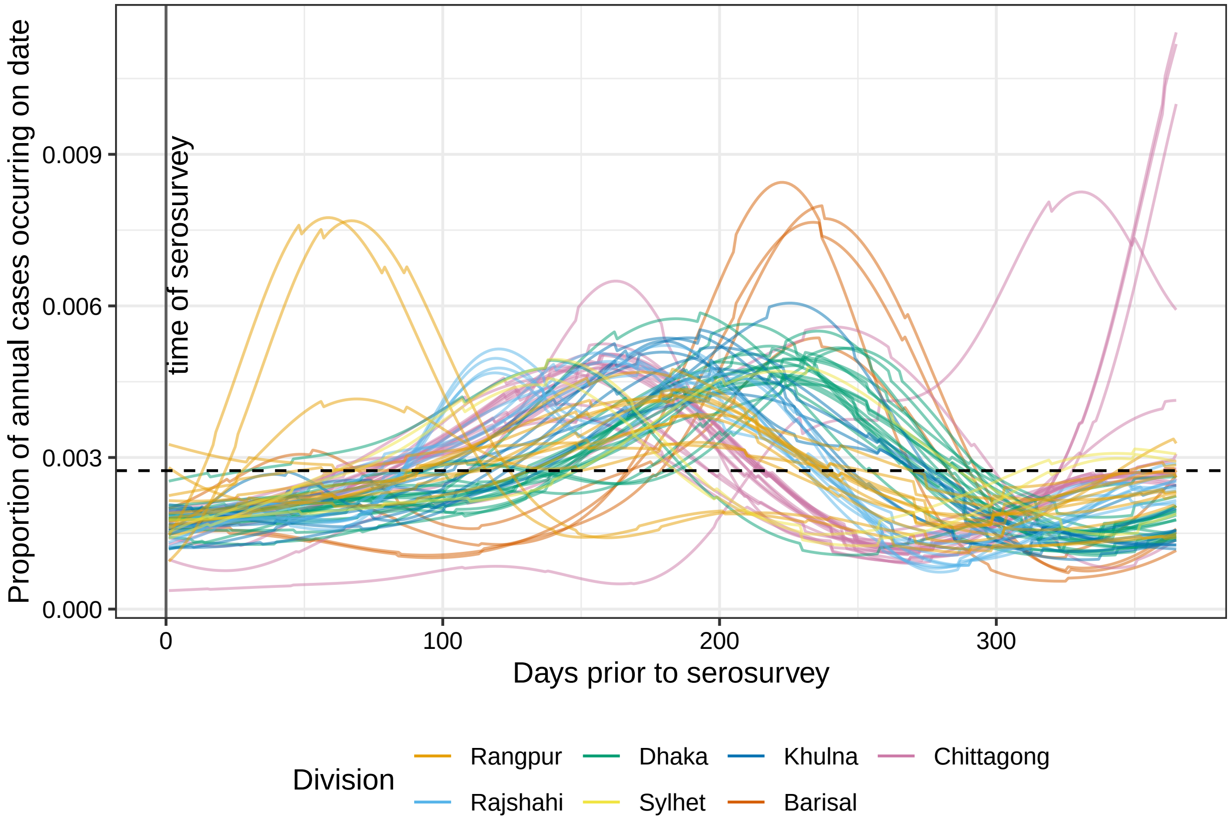

Figure S2: The estimated daily proportion of annual cholera cases by days prior to the serosurvey for each sampled community. These estimates are based on sentinel surveillance data and reported numbers of acute watery diarrhea by each division in Bangladesh. Each solid line shows a different sampled community, with the color determined by the division the community resides in. The dashed black line shows the probability if all days had the same proportion (1/365).

To make seroincidence estimates incorperating seasonality of infection risk, we use these $\mathbb{P}(T = t \mid Y = 1, C = c)$ in place of $\mathbb{P}(T = 1 \mid Y = 1)$ in a slightly modified version of the Stan model.

S1.5. Other estimators and time frames

We fit several alternative models and observe the differences in their resulting seroincidence estimates. We use an 'unadjusted' model, where our random forest estimates are not adjusted by sensitivity and specificity (i.e. $\pi_i\theta^{1|1} + (1 - \pi_i)(1 - \theta^{0|0})$ in Equation 1 is replaced by π_i). Previous work showed that a vibriocidal titer (either Inaba and Ogawa) of at least 320 was the best threshold for maximimizing sensitivity and specificity for identifying individuals infected in the previous year; thus we fit a 'vibriocidal' model which used these predictions in place of the random forest predictions for z_i in Equation 1. To see how the seroincidence changed over multiple time frames, we also fit the random forest and vibriocidal models to 100 and 200 days infection windows. We use the raw aggregated random forest predictions to make the unadjusted estimate and use the same Stan framework as in the main analysis to estimate the seroincidence using the vibriocidal cutoff and for other time windows.

S1.6. Risk factors for seropositivity

We used a series of logistic regression models with a Matern spatial covariance function to explore the association between seropositivity (random forest positive for individuals) and various individual-, household- and community-level covariates. We explored both univariate relationship and multivariate (linear) relationships between the covariates and the binary seropositivity outcome using models with and without different random effects and spatial correlation. The 'full' model, used for the primary analyses in manuscript, included a Matern spatial random field and random effects for both households and communities (assumed to be independent and identically distributed with log-gamma priors). We also estimated the relationship between the covariates and seropositivity with a model including no random effects for household or community and only spatial correlation, and another model including only random effects for household and community without spatial correlation.

S1.7. Results

The three methods produced similar estimates for the nationwide seroincidence rate for the 365-day infection window before the serosurvey (Figure S3A). The median spatial estimate from the INLA 5km by 5km grid-cell maps was 17.3% (95% CI: 10.5-24.1%) This corresponds to a median of 28.1 million (95% CI: 17.1-39.2 million) individuals infected during that time period. The median post-stratified estimate from the Bayesian hierarchical model, $\pi^{poststrat}$ in Equation 10, was 19.9% (95% CI: 15.1-25.0%). The median in-sample estimate from the same model, π^{survey} in Equation 6, was 20.3% (95% CI: 15.7-25.3%). While the median is a little lower for the spatial estimate, all of the 95% credible intervals are overlapping.

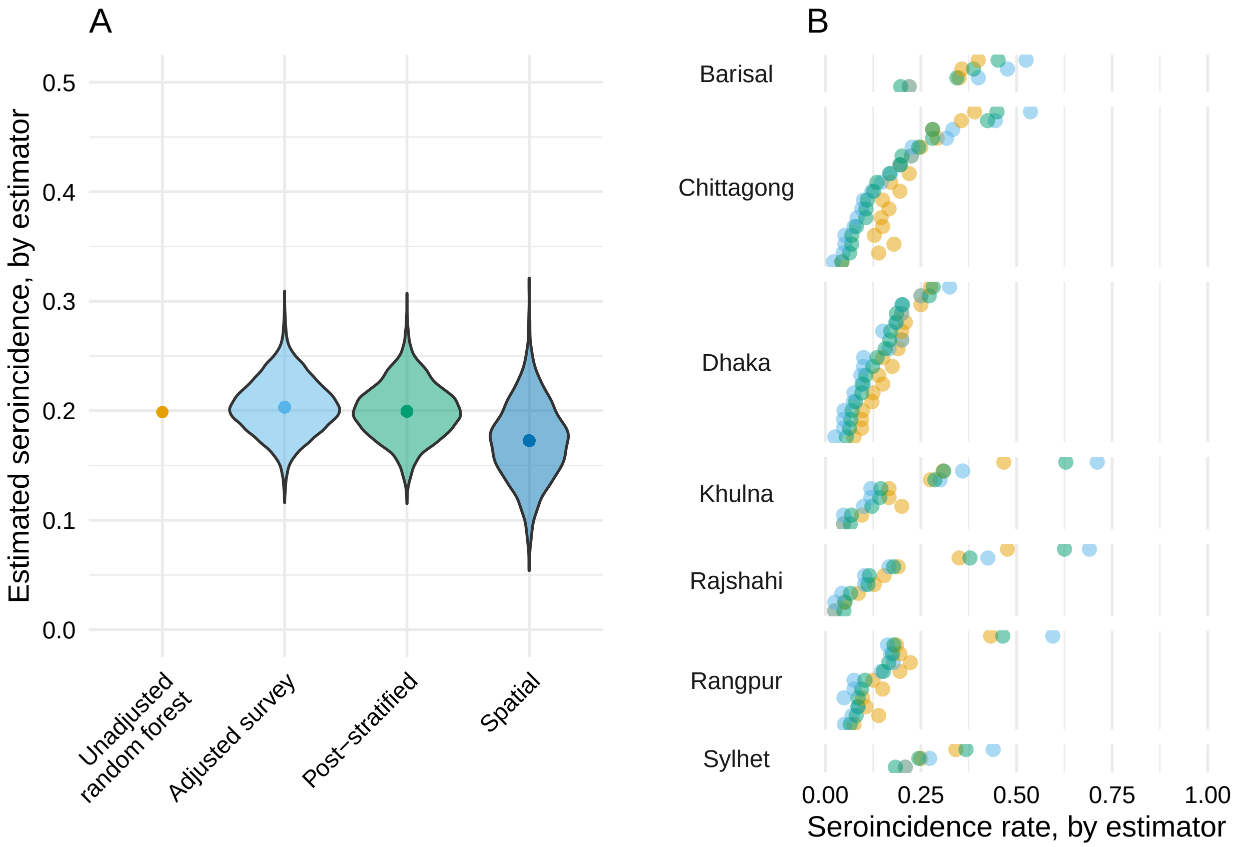

Figure S3: The estimated annual seroincidence rate medians and distributions by estimator for the whole population (A) and by community (B). The unadjusted random forest estimates (orange) do not account for sensitivity or specificity. The adjusted survey estimates (light blue) are the survey estimates from the Bayesian hierarchical model which accounts for the sensitivity and specificity, of the random forest estimates, as well as age, sex, and the community population size. The post-stratified estimates (green) are from the same model but extrapolating to the unsampled population in each community based on its demographics. The spatial estimates (dark blue) extend the survey estimates to the rest of the country using a logistic regression with a Matern spatial covariance function. There are no community-specific estimates for the spatial model in (B).

S1.7.1. Alternate analyses

The unadjusted 365-day random forest model nationwide estimate is similar to the median estimates from the adjusted and post-stratified models (median: 19.9%), however there is considerable variability between locations (Figure S3B). By comparison, the vibriocidal model yields lower seroincidence rate estimates (median: 15.0%, 95% CI: 10.8-19.6%) than the models based on random forest estimates despite the fact that the proportion of the serosurvey that had vibriocidal titers greater than or equal to 320 is similar to the proportion that was classified as seropositive by the random forest model (19.5% vs. 19.9%). This is due to the vibriocidal estimates having a lower specificity than the random forest models (Figure S4).

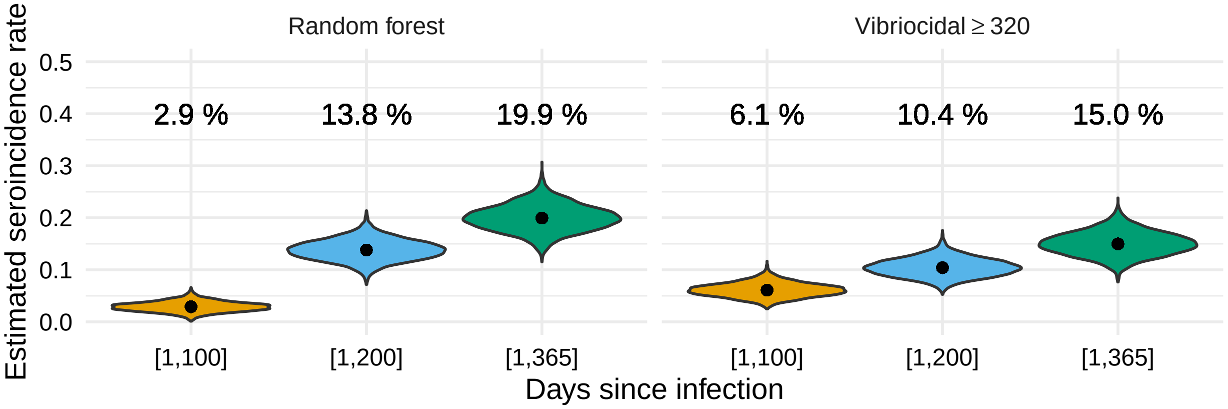

	RF [1,100]	RF [1,200]	RF [1,365]	Vib320 [1,100]	Vib320 [1,200]	Vib320 [1,365]
sensitivity	67.9%	65.0%	55.4%	74.3%	63.5%	53.3%
specificity	93.8%	89.7%	89.0%	82.3%	84.8%	86.0%

Figure S4: The estimated seroincidence rate across varying infection window sizes and estimators. We estimate the seroincidence rate with two different estimators across three infection time windows (100, 200, and 365 days). The random forest model (RF) uses age, sex, and measurements of six antibodies (vibriocidal Inaba, vibriocidal Ogawa, anti-CTB IgG, anti-CTB IgA, anti-LPS IgG and anti-LPS IgA) to classify individuals as seroincident. As a comparison, we use the historical convention where those with either vibriocidal titers greater than or equal to 320 is classified as seroincident. The vibriocidal titer method has lower specificity, which yields lower estimates of seropositivity than those from the random forest model. The estimate of the median seroincidence rate with each estimator is displayed above its distribution. The estimates of the adjusted sensitivity and specificity for each estimator and window size are presented in the table below the figure.

The model including seasonality produced overall post-stratified estimates that were very similar to the main estimates (median: 20.1%, 95% CI: 15.5-24.6%). The community-level estimates were also similar, with the largest difference between the estimates in any community being 4.1%. 80.0% of estimates accounting for seasonality of risk were within 1% of estimates not accounting for seasonality (Figure S5). Estimates adjusted for seasonality were also similar to the main estimates at 100 and 200 days.

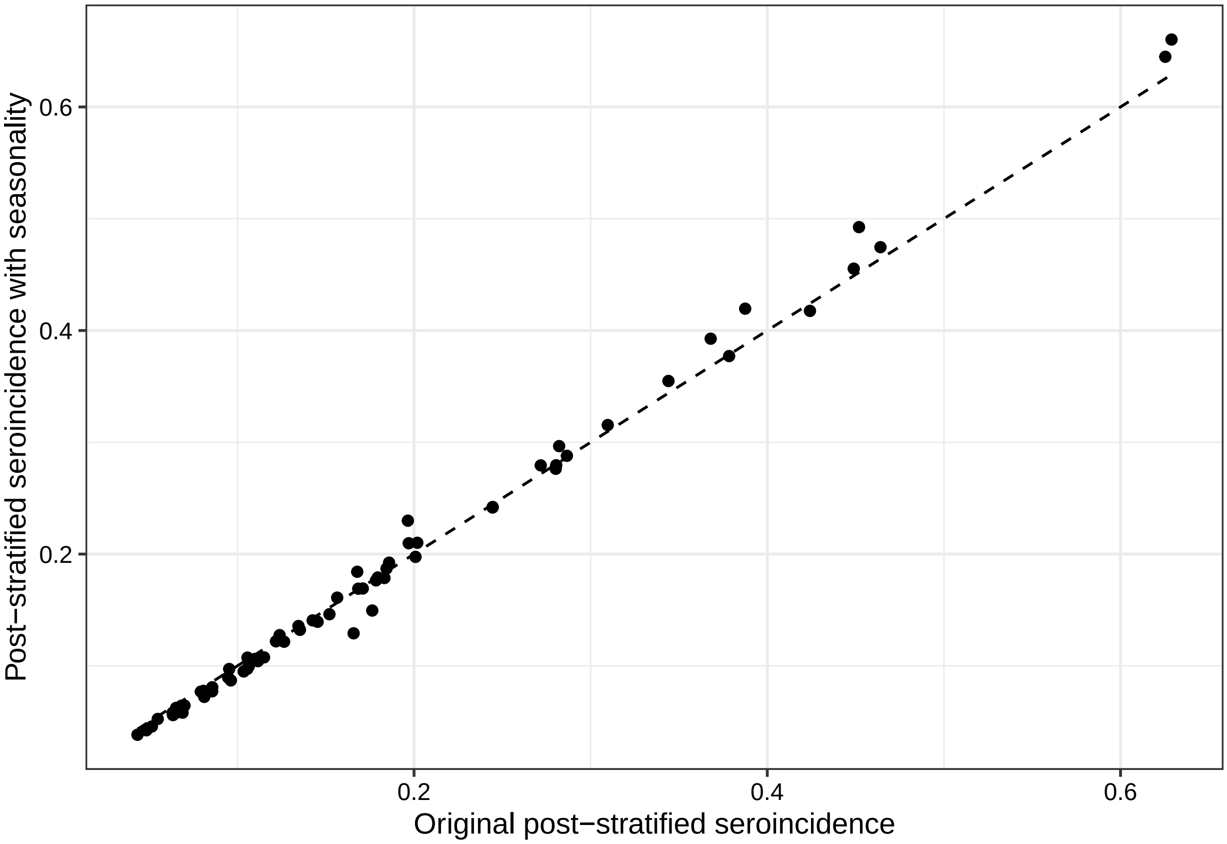

Figure S5: Comparison of post-stratified seroincidence estimates with and without seasonality by community.

S1.7.2. LOOCV

As described above, to choose the covariates for the model to make the spatial maps, we used a backwards-stepwise selection using WAIC. The model that minimized the WAIC had two covariates, the proportion of the grid cell that is female and poverty. We compared this model to a null model that had a spatial field but no linear covariates and a naive model that used the average of all other communities using leave-one-community-out cross validation and evaluating their predictions using mean absolute error (MAE). The null model had the least error (MAE: 8.5%) and was similar to the model with covariates (MAE: 8.5%); both had smaller MAE than the naive model (MAE: 9.8%), thus we used this null model in our primary analyses. Figure S6 shows a comparison of the community-specific results.

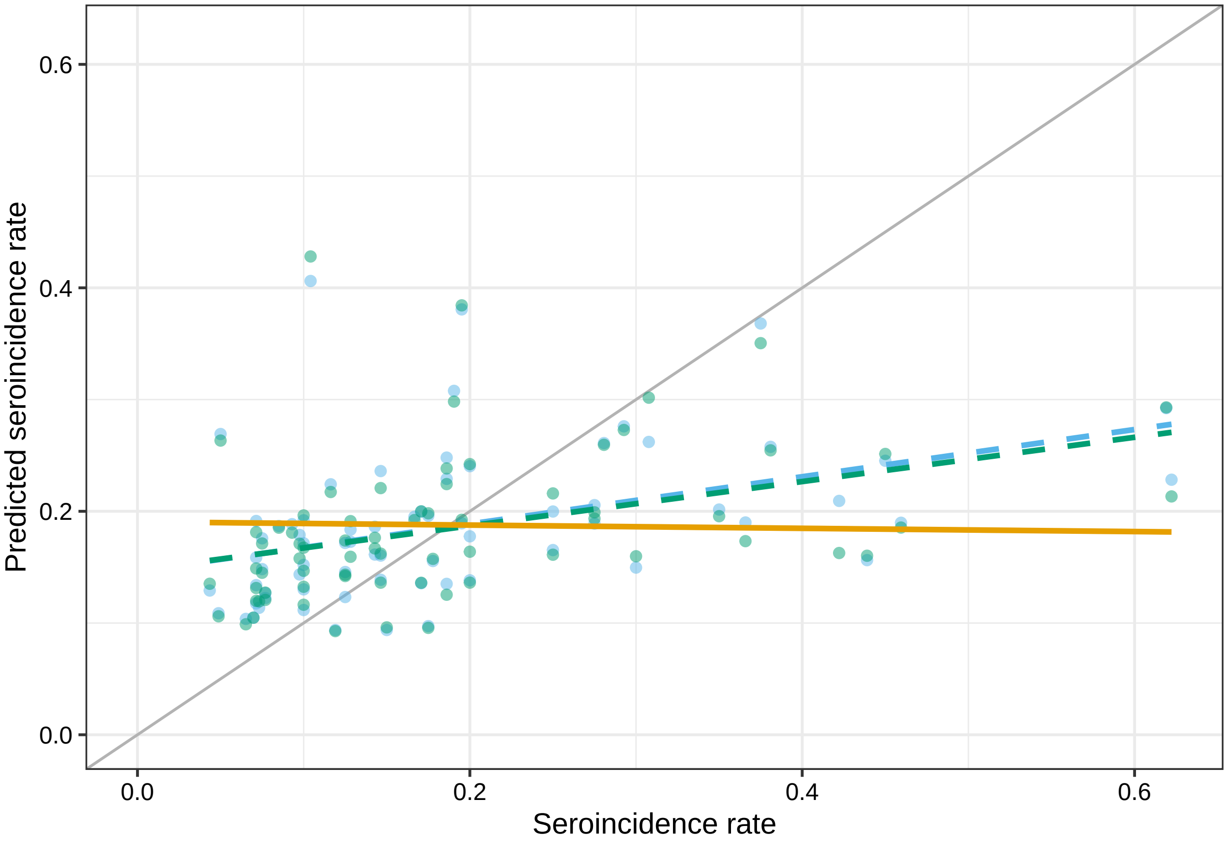

Figure S6: The results from leave-one-community-out cross validation. Results from cross-validation where each community was held out of the INLA model, one at a time, and the posterior predictive mean for that location was estimated (y-axis). The green dashed line illustrates the best fit line for predictions from the model with proportion female and poverty as covariates. The blue dashed line illustrates the best fit line for the null model predictions. The solid orange line is the best fit line for a naive model that predicts the average of the mean of the other sampled communities.

S1.7.3. Mapping

Using the null model (only spatial random field and no covariates), we made grid-cell estimates of seroincidence for all of Bangladesh. From these we made a series of maps to observe the geographic variability of cholera throughout Bangladesh. Maps of the median seroincidence rate and estimated number of annual infections by grid cell are in the main manuscript (Figure 2). To help identify high-risk regions and our confidence in the estimates, we calculated proportion of posterior grid-cell seroincidence estimates with a relative risk greater than two (Figure S7). As in the manuscript, we see higher risk in the Bay of Bengal and in pockets in the northwest and north, though less so in the northeast.

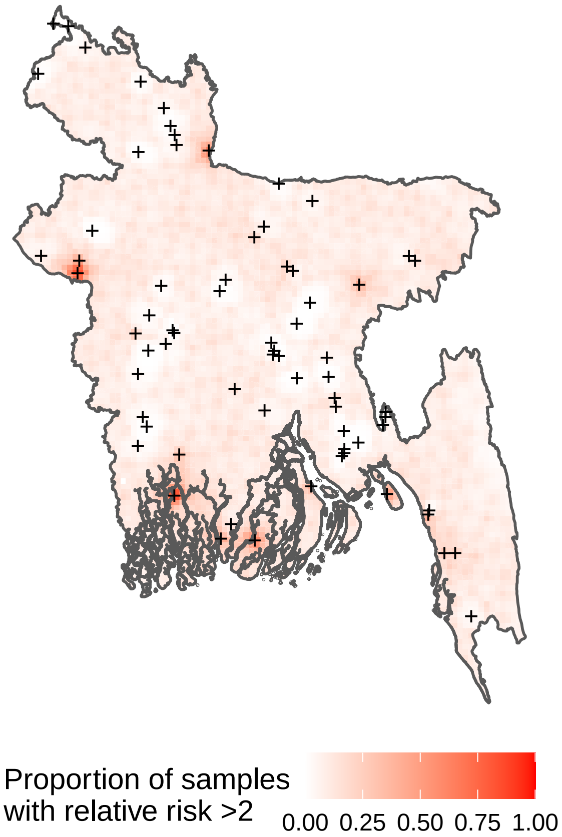

Figure S7: Proportion of posterior samples with relative risk greater than 2 for each 5km x 5km grid cell.

Figure S8 investigates the variance of our estimates. As variance scales with the size of the estimate, it is difficult to interpret. The coefficient of variation, the standard deviation divided by the mean, is another measure often used but it can become very large for places where mean estimates are very small. Instead, we use the width of the logged relative risk credible interval in this map. To do this, we first bound all posterior samples of the relative risk to be between 0.25 and 4 (or -2 and 2 on the log_2 scale), which represent reasonable cutoffs for very high and very low risk as only 2.1% of upper bounds across all grid cells are greater than 4, though 93.4% of lower bounds are less than 0.25. Next, we take the log_2 difference between the upper and lower bounds of the 95% credible interval for each grid cell. Grid cells with a log_2 difference of 4 have an upper bound relative risk that is greater than 4 and a lower bound relative risk less than 0.25, indicating that we are very uncertain of the true risk in that grid cell; 1.0% of the grid cells in our map have a log_2 difference of 4 and are displayed in white. Grid cells with a log_2 difference of 0 have both upper and lower bounds either above 4 or below 0.25 and would be indicated on the map with maximum opacity if there were any. The colors on the map indicate the posterior median for that grid cell and the opacity indicates the log_2 difference between the upper and lower bounds. This map demonstrates how the certainty in our estimates fades with distance from the sampled communities.

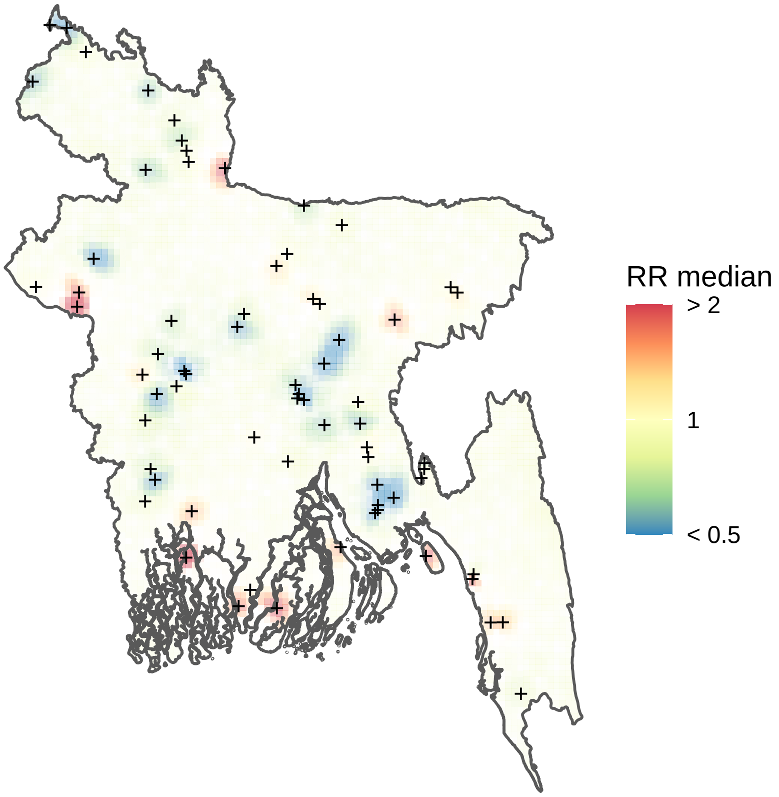

Figure S8: The median relative risk estimate for each 5km x 5km grid cell with the opacity determined by the width of the 95% credible interval. White grid cells have 95% credible intervals where the lower bound is less than 0.25 and the upper bound is greater than 4. Places with narrower credible intervals have greater opacity. The most opaque cells are those where both the upper and lower bound are either less than 0.25 or above 4.

S1.8. District-level estimates

Table S2: Seroincidence estimates for the districts of Bangladesh. Note that these are classified by administrative divisions using GADM 3.0, which does not reflect changes in administrative divisions after late 2015.

Division	District	Seroincidence rate, % (median, 95% CI)	Implied infections, ’000s (median, 95% CI)	Relative risk (median, 95% CI)	Proportion of total, % (median, 95% CI)
Barisal	Barisal	17.5 (5.7-35.4)	468 (154-949)	1.00 (0.41-1.88)	1.7 (0.7-3.1)
	Bhola	18.9 (8.9-36.3)	372 (175-713)	1.10 (0.58-1.98)	1.3 (0.7-2.4)
	Borgona	25.7 (12.2-45.9)	272 (130-487)	1.47 (0.79-2.75)	1.0 (0.5-1.8)
	Jhalakati	16.7 (4.1-44.9)	113 (28-305)	0.98 (0.28-2.32)	0.4 (0.1-1.0)
	Patuakhali	19.5 (8.0-40.1)	329 (135-676)	1.12 (0.58-2.19)	1.2 (0.6-2.3)
	Pirojpur	20.2 (9.1-37.2)	287 (129-529)	1.17 (0.59-2.08)	1.0 (0.5-1.8)
	Bandarbon	17.2 (7.5-32.7)	81 (36-155)	1.00 (0.53-1.73)	0.3 (0.2-0.5)
	Brahmanbaria	17.4 (5.9-38.5)	564 (192-1,243)	1.01 (0.43-2.01)	2.0 (0.9-4.0)
	Chandpur	15.7 (5.1-34.3)	437 (143-958)	0.91 (0.35-1.90)	1.6 (0.6-3.3)
	Chittagong	20.2 (10.4-35.2)	1,642 (848-2,861)	1.16 (0.75-1.84)	5.8 (3.7-9.2)

Chittagong	Comilla	15.1 (6.0-26.6)	922 (367-1,629)	0.87 (0.42-1.46)	3.3 (1.6-5.5)
	Cox's Bazar	17.0 (5.9-33.2)	460 (160-901)	0.98 (0.42-1.85)	1.6 (0.7-3.1)
	Feni	15.4 (4.9-32.6)	252 (79-532)	0.89 (0.32-1.89)	0.9 (0.3-1.9)
	Khagrachari	16.8 (6.6-34.2)	115 (45-232)	0.97 (0.43-1.87)	0.4 (0.2-0.8)
	Lakshmipur	15.7 (4.8-33.7)	309 (94-664)	0.89 (0.34-1.88)	1.1 (0.4-2.3)
	Noakhali	14.8 (5.8-27.9)	530 (207-997)	0.86 (0.43-1.51)	1.9 (0.9-3.3)
	Parbattya Chattagram	16.9 (7.4-30.7)	115 (50-209)	0.98 (0.55-1.60)	0.4 (0.2-0.7)
Dhaka	Dhaka	14.4 (4.5-28.4)	1,973 (610-3,892)	0.82 (0.32-1.52)	7.0 (2.7-12.8)
	Faridpur	16.9 (6.0-35.7)	367 (130-774)	1.00 (0.40-1.91)	1.3 (0.5-2.6)
	Gazipur	14.6 (4.0-33.4)	505 (138-1,157)	0.85 (0.26-1.88)	1.8 (0.6-4.0)
	Gopalgonj	17.2 (5.3-37.7)	240 (74-526)	1.02 (0.36-2.07)	0.9 (0.3-1.8)
	Jamalpur	18.7 (6.6-40.5)	483 (170-1,046)	1.06 (0.49-2.26)	1.7 (0.8-3.6)
	Kishoreganj	17.2 (6.4-35.4)	553 (205-1,140)	0.99 (0.47-1.89)	2.0 (0.9-3.8)
	Madaripur	16.8 (4.8-37.9)	219 (62-494)	0.97 (0.31-2.16)	0.8 (0.3-1.7)
	Manikgonj	16.0 (3.4-37.5)	252 (53-589)	0.92 (0.25-2.02)	0.9 (0.2-2.0)
	Munshigonj	14.0 (3.2-32.7)	230 (53-538)	0.82 (0.23-1.83)	0.8 (0.2-1.9)
	Naray Angonj	12.2 (2.7-31.0)	418 (93-1,063)	0.72 (0.20-1.77)	1.5 (0.4-3.7)
	Narshingdi	11.1 (2.8-26.0)	281 (70-656)	0.65 (0.20-1.44)	1.0 (0.3-2.2)
	Nasirabad	18.2 (8.5-31.2)	1,028 (479-1,761)	1.04 (0.63-1.64)	3.6 (2.2-5.7)
	Netrakona	16.1 (6.3-31.5)	408 (161-798)	0.94 (0.40-1.73)	1.5 (0.6-2.7)
	Rajbari	15.0 (4.5-32.3)	179 (54-385)	0.87 (0.32-1.81)	0.6 (0.2-1.3)
	Shariatpur	15.9 (4.9-34.2)	214 (66-460)	0.92 (0.35-1.88)	0.8 (0.3-1.6)
	Sherpur	17.4 (4.9-40.2)	259 (73-597)	0.99 (0.37-2.23)	0.9 (0.3-2.0)
	Tangail	15.6 (5.8-30.5)	644 (239-1,261)	0.90 (0.41-1.63)	2.3 (1.0-4.1)
Khulna	Bagerhat	20.7 (9.0-40.5)	317 (137-619)	1.18 (0.59-2.21)	1.1 (0.6-2.1)
	Choua Danga	16.6 (4.5-40.8)	215 (59-530)	0.95 (0.28-2.30)	0.8 (0.2-1.8)
	Jessore	14.7 (5.5-27.5)	466 (172-868)	0.85 (0.38-1.52)	1.6 (0.7-3.0)
	Jhenaidah	15.0 (4.6-28.8)	302 (93-580)	0.85 (0.33-1.55)	1.1 (0.4-1.9)
	Khulna	22.0 (11.5-39.6)	591 (309-1,064)	1.29 (0.79-2.12)	2.1 (1.3-3.5)
	Kustia	17.9 (6.7-33.5)	395 (148-740)	1.03 (0.46-1.83)	1.4 (0.6-2.5)
	Magura	15.0 (3.7-37.4)	156 (39-391)	0.87 (0.25-2.09)	0.6 (0.2-1.3)
	Meherpur	16.0 (3.9-42.9)	124 (30-332)	0.92 (0.29-2.46)	0.4 (0.1-1.2)
	Narail	16.3 (4.5-39.4)	132 (36-319)	0.94 (0.31-2.16)	0.5 (0.2-1.1)
	Shatkhira	17.5 (6.7-35.0)	419 (160-839)	0.99 (0.51-1.82)	1.5 (0.8-2.7)
	Bogra	17.2 (6.2-34.8)	655 (238-1,323)	0.98 (0.43-1.87)	2.3 (1.0-4.4)
	Jaipurhat	15.9 (3.6-37.9)	161 (37-384)	0.91 (0.25-2.03)	0.6 (0.2-1.3)
	Naogaon	15.2 (5.8-30.6)	449 (170-901)	0.91 (0.39-1.58)	1.6 (0.7-2.9)
	Natore	17.3 (4.8-37.2)	335 (94-721)	0.99 (0.38-2.16)	1.2 (0.4-2.6)

Rajshahi	Nawabganj	17.4 (6.1-33.8)	331 (117-643)	0.98 (0.37-1.90)	1.2 (0.4-2.2)
	Pabna	13.4 (4.6-26.6)	390 (132-773)	0.78 (0.33-1.45)	1.4 (0.6-2.6)
	Rajshahi	23.5 (12.1-43.3)	686 (353-1,262)	1.36 (0.80-2.59)	2.4 (1.4-4.7)
	Sirajgonj	16.3 (4.3-32.5)	564 (150-1,127)	0.92 (0.32-1.74)	2.0 (0.7-3.7)
Rangpur	Dinajpur	16.4 (5.8-32.5)	549 (193-1,088)	0.94 (0.41-1.73)	1.9 (0.8-3.6)
	Gaibanda	17.6 (7.0-36.3)	466 (184-961)	1.02 (0.47-1.91)	1.7 (0.8-3.1)
	Kurigram	19.4 (7.5-35.1)	449 (174-812)	1.10 (0.51-2.15)	1.6 (0.7-3.1)
	Lalmonirhat	14.7 (4.4-31.2)	202 (60-427)	0.87 (0.30-1.73)	0.7 (0.2-1.5)
	Nilphamari	16.6 (4.8-36.8)	337 (98-747)	0.95 (0.34-1.94)	1.2 (0.4-2.4)
	Panchagarh	15.0 (4.6-30.3)	164 (50-330)	0.87 (0.32-1.63)	0.6 (0.2-1.1)
	Rongpur	14.8 (4.9-28.6)	480 (158-930)	0.85 (0.33-1.64)	1.7 (0.7-3.3)
	Thakurgaon	14.7 (4.2-31.3)	229 (66-487)	0.84 (0.27-1.72)	0.8 (0.3-1.7)
Sylhet	Hobiganj	18.6 (7.5-38.6)	435 (175-905)	1.07 (0.52-2.01)	1.5 (0.7-2.9)
	Moulvibazar	18.0 (7.3-35.3)	385 (155-755)	1.04 (0.53-1.92)	1.4 (0.7-2.5)
	Sun Amgonj	17.2 (6.2-35.2)	485 (174-989)	0.99 (0.43-1.79)	1.7 (0.7-3.1)
	Sylhet	17.1 (6.1-34.6)	655 (233-1,322)	0.98 (0.44-1.84)	2.3 (1.0-4.3)

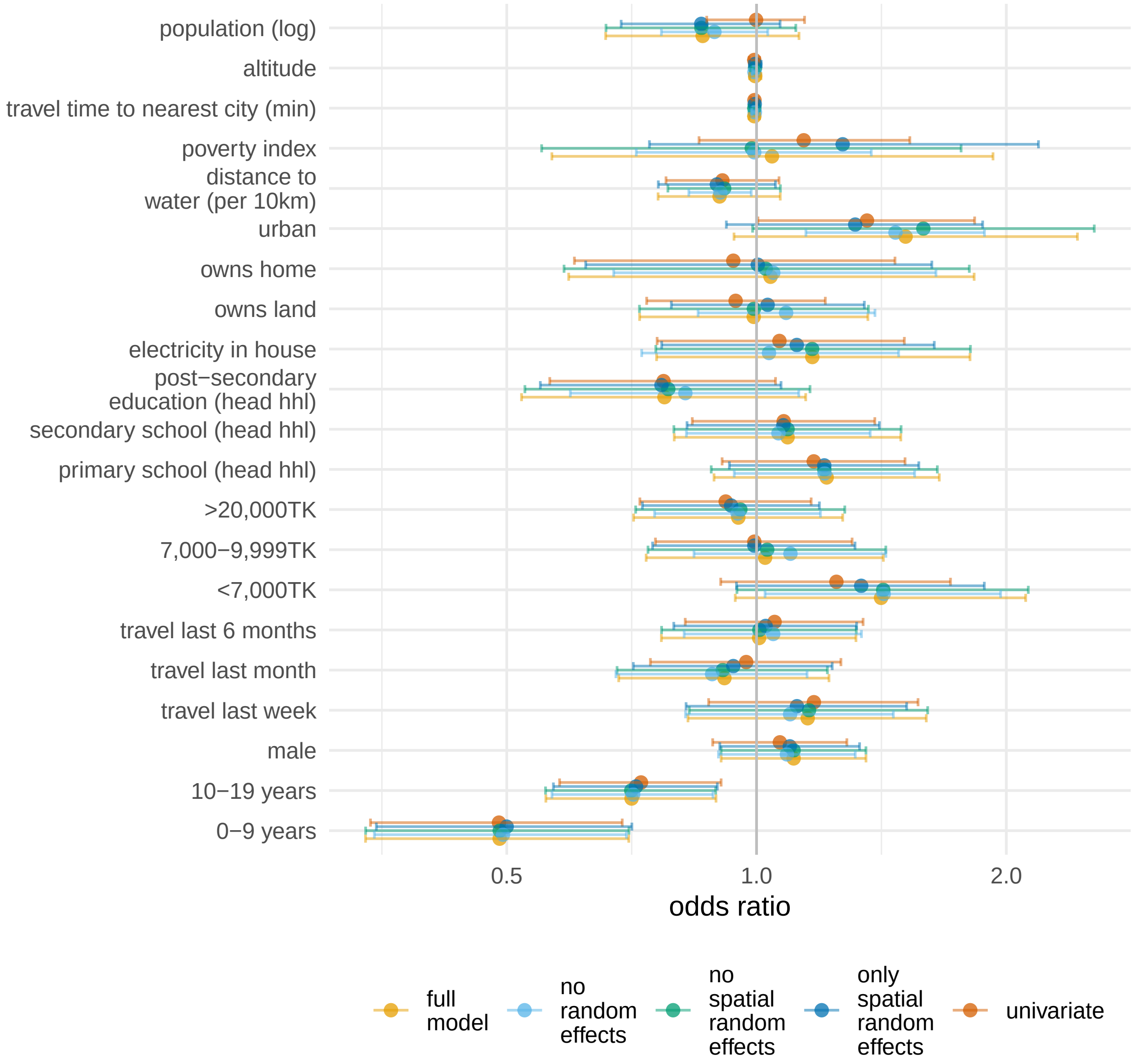

Figure S9: Estimates of odds ratios for seropositivity from different models.

S2. Additional descriptive analyses and figures

In this section we present additional descriptive analyses to illustrate the distributions of each of the antibody levels in different ways and characteristics of the cohort.

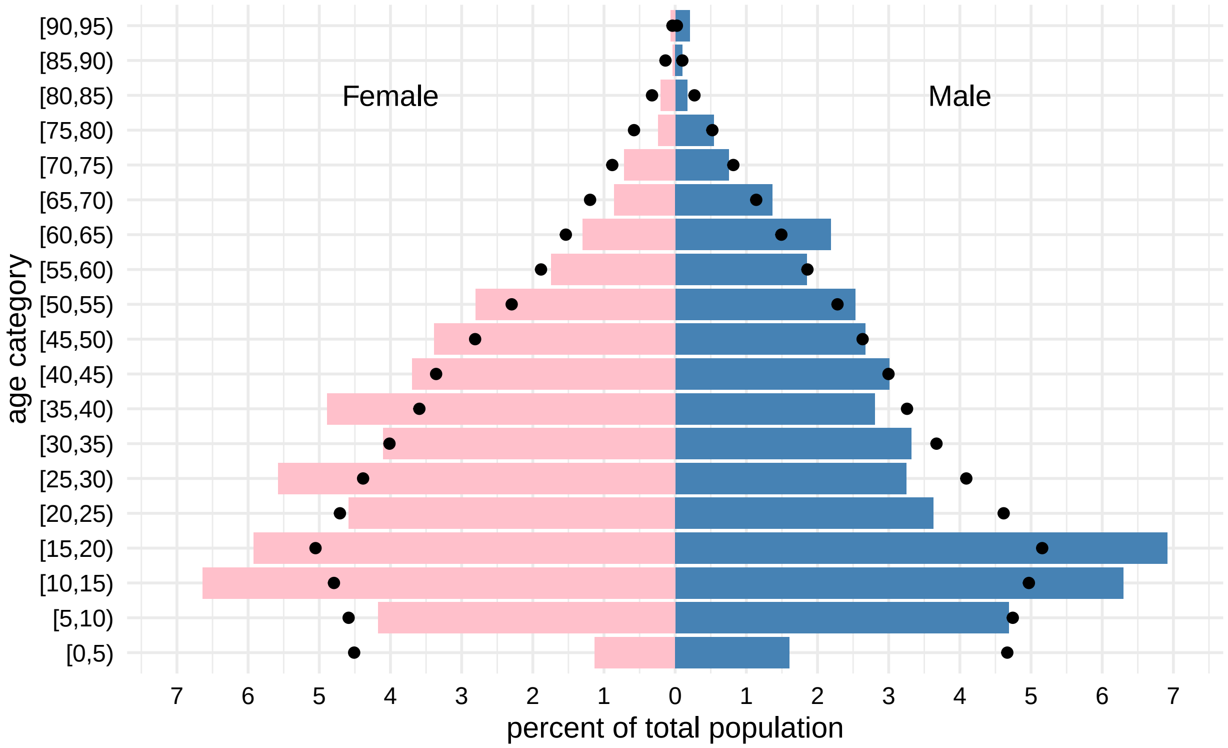

Figure S10: Population pyramid of survey participants. Dots illustrate the expected proportion of each age-sex category according to the 2012 Bangladesh census.

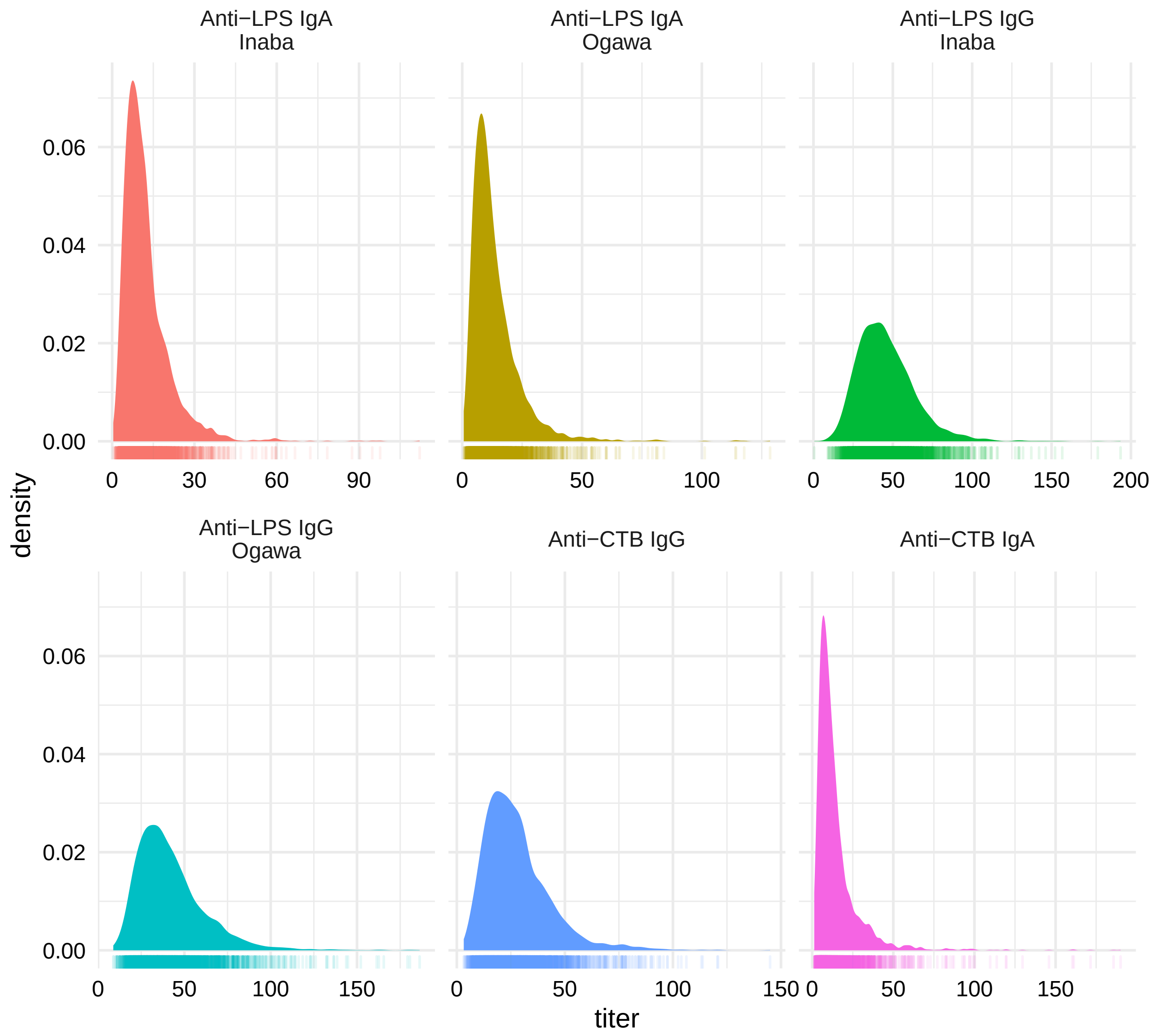

Figure S11: Distributions (smoothed) of antibodies measured by ELISA. Smoothed densities estimated using ggplot with default parameters (geom_density) with locations of data points shown in the rug plot below.

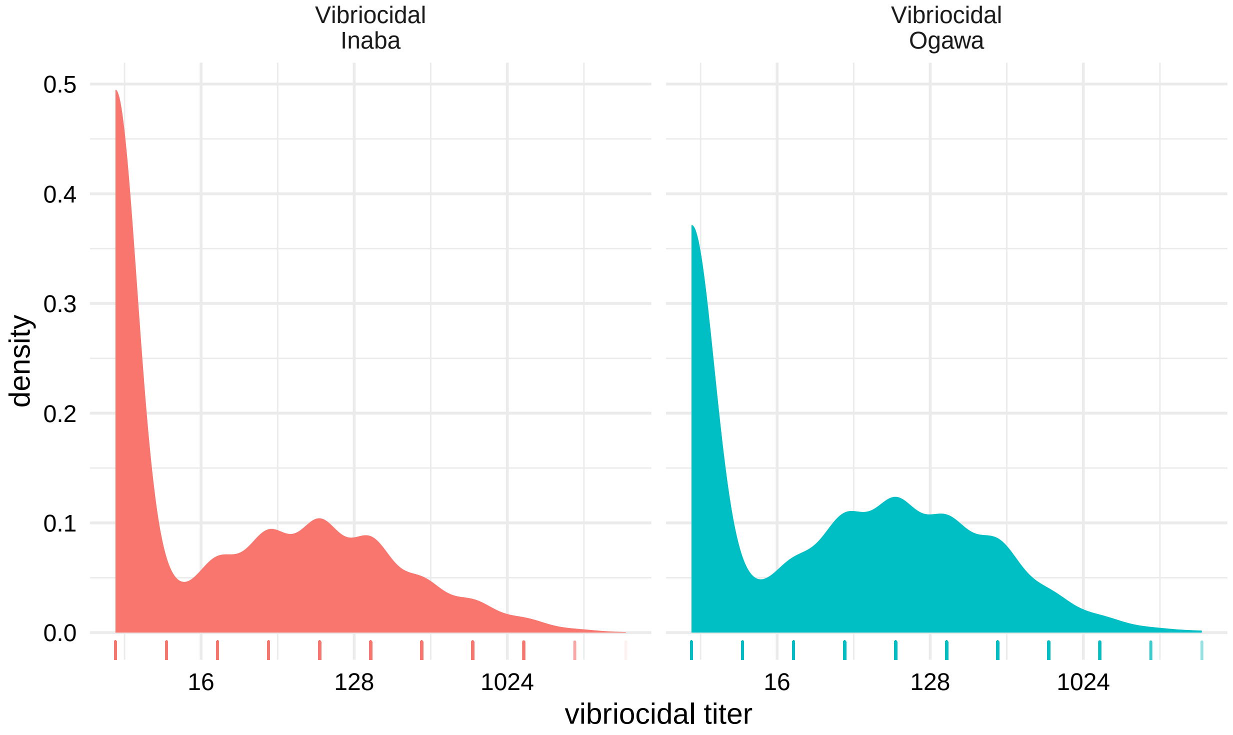

Figure S12: Distributions (smoothed) of vibriocidal antibodies. Smoothed densities estimated using ggplot with default parameters (geom_density) with locations of data points shown in the rug plot below.

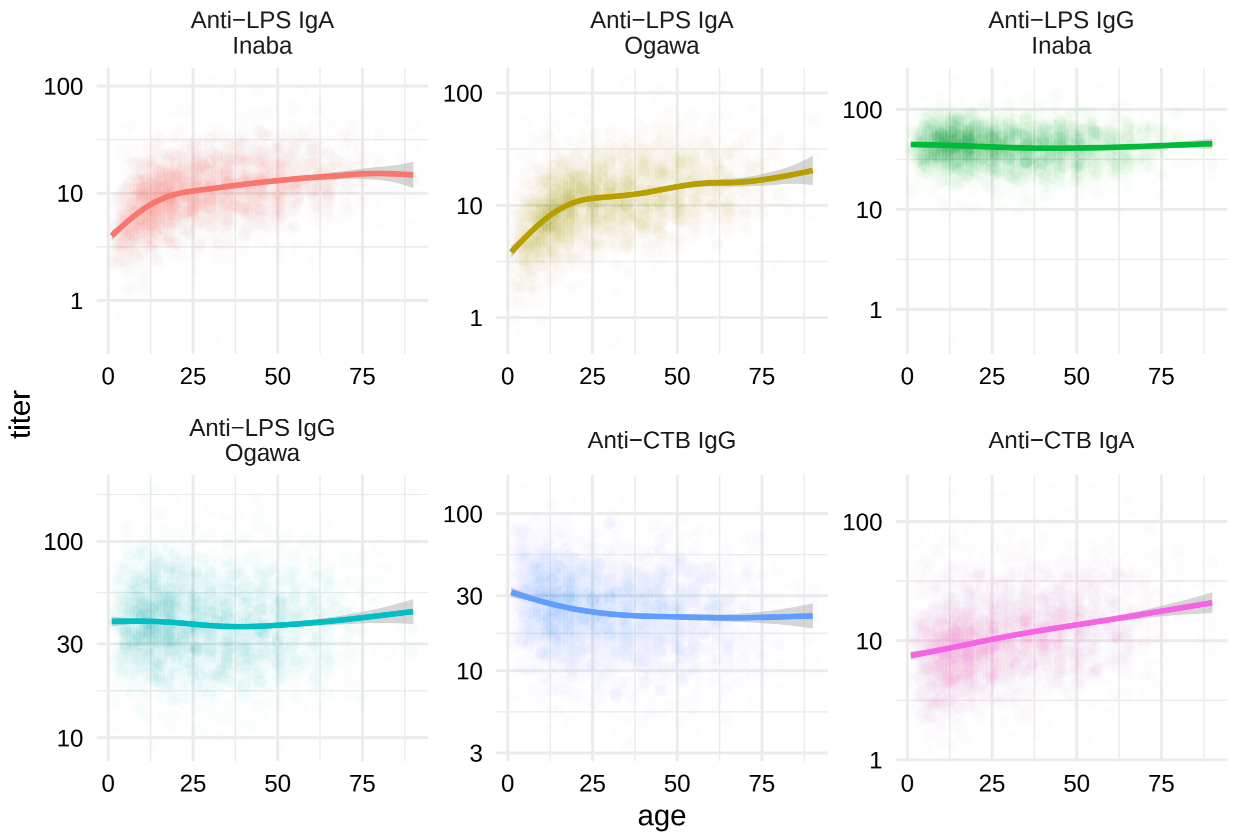

Figure S13: Distributions of ELISA antibody titers by age. Dots represent individual datapoints and lines represent the fit of a generalized additive model using a cubic spline.

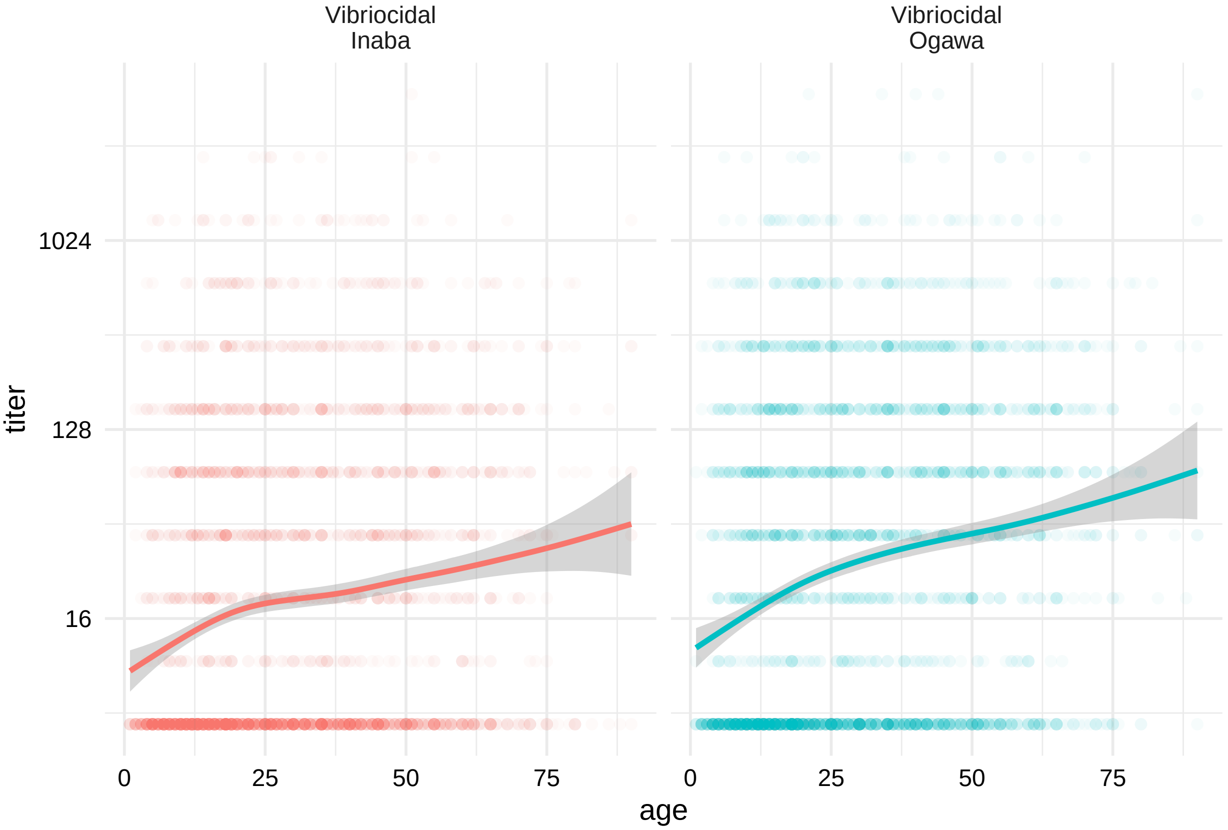

Figure S14: Distributions of vibriocidal antibody titers by age. Dots represent individual datapoints and lines represent the fit of a generalized additive model using a cubic spline.

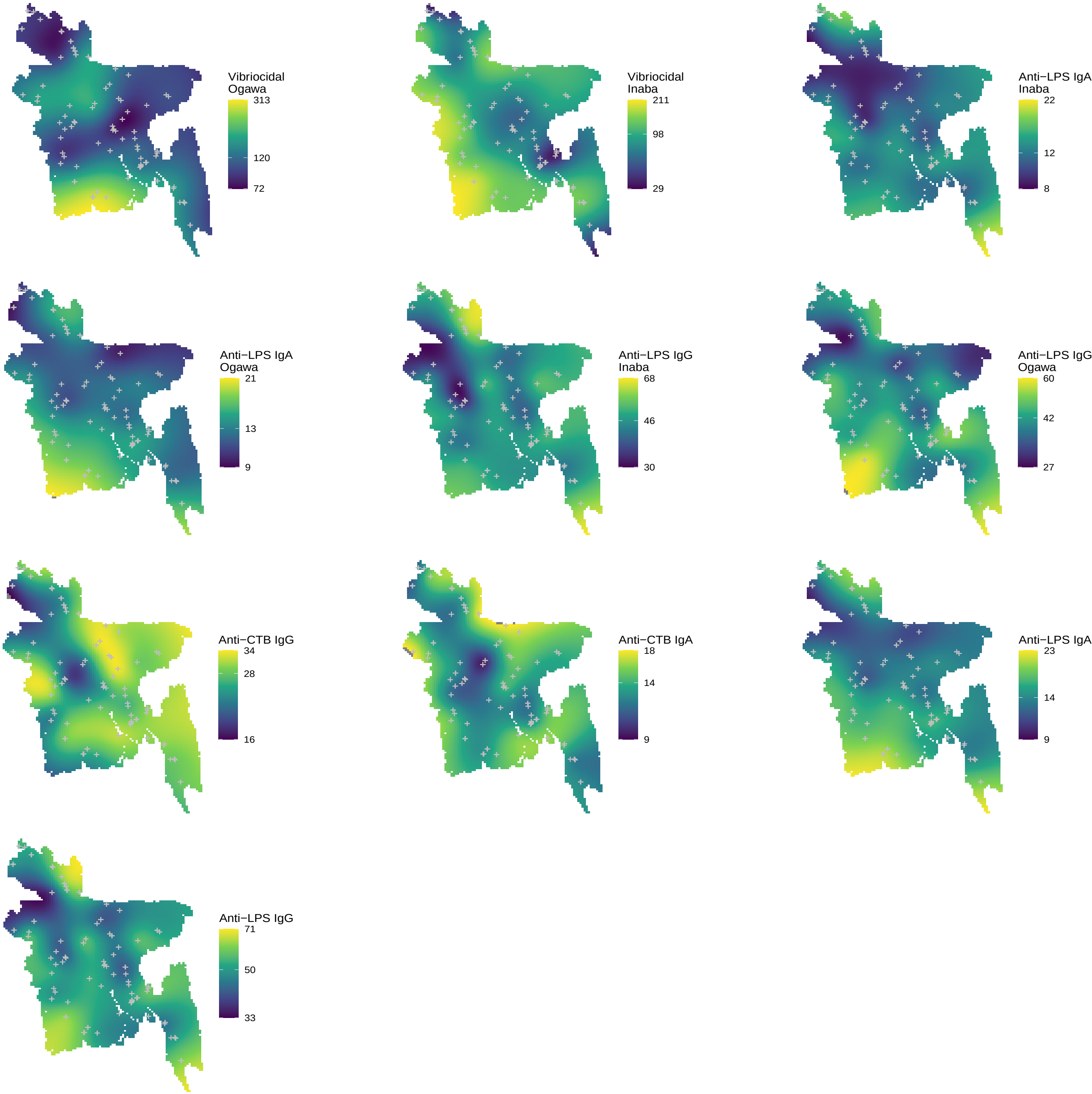

Figure S15: Smoothed maps of the antibody levels for each biomarker based on generalized additive models (GAMs) including a thinplate spline for geographic coordinates and age. Predictions are made for individuals of age 25 to reflect that of adults.

References

[1] Makela S, Si Y, Gelman A. Bayesian inference under cluster sampling with probability proportional to size. Statistics in Medicine 2018;37:3849–68. doi:10.1002/sim.7892.

[2] Carpenter B, Gelman A, Hoffman MD, Lee D, Goodrich B, Betancourt M, et al. Stan: A Probabilistic Programming Language. Journal of Statistical Software 2017;76:1–32. doi:10.18637/jss.v076.i01.

[3] Stan Development Team. RStan: The R interface to Stan 2018.

[4] Rue H, Martino S, Chopin N. Approximate Bayesian inference for latent Gaussian models by using integrated nested Laplace approximations. Journal of the Royal Statistical Society: Series B (Statistical Methodology) 2009;71:319–92. doi:10.1111/j.1467-9868.2008.00700.x.

[5] Azman AS, Lessler J, Luquero FJ, Bhuiyan TR, Khan AI, Chowdhury F, et al. Estimating cholera incidence with cross-sectional serology. Science Translational Medicine 2019;11:eaau6242. doi:10.1126/scitranslmed.aau6242.

[6] Youden WJ. Index for rating diagnostic tests. Cancer 1950;3:32–5. doi:10.1002/1097-0142(1950)3:1<32::AID-CNCR2820030106>3.0.CO;2-3.

[7] Leisenring W, Pepe MS, Longton G. A Marginal Regression Modelling Framework for Evaluating Medical Diagnostic Tests. Statistics in Medicine 1997;16:1263–81. doi:10.1002/(SICI)1097-0258(19970615)16:11<1263::AID-SIM550>3.0.CO;2-M.

[8] Das SK, Begum D, Ahmed S, Ferdous F, Farzana FD, Chisti MJ, et al. Geographical diversity in seasonality of major diarrhoeal pathogens in Bangladesh observed between 2010 and 2012. Epidemiology & Infection 2014;142:2530–41. doi:10.1017/S095026881400017X.

[9] Khan AI, Rashid MM, Islam MT, Afrad MH, Salimuzzaman M, Hegde ST, et al. Epidemiology of Cholera in Bangladesh: Findings From Nationwide Hospital-based Surveillance, 2014-2018. Clinical Infectious Diseases: An Official Publication of the Infectious Diseases Society of America 2019. doi:10.1093/cid/ciz1075.
